# Structure-based discovery of mPGES-1 inhibitors suitable for preclinical testing in wild-type mice as a new generation of anti-inflammatory drugs

**DOI:** 10.1038/s41598-018-23482-4

**Published:** 2018-03-26

**Authors:** Kai Ding, Ziyuan Zhou, Shurong Hou, Yaxia Yuan, Shuo Zhou, Xirong Zheng, Jianzhong Chen, Charles Loftin, Fang Zheng, Chang-Guo Zhan

**Affiliations:** 1 Molecular Modeling and Biopharmaceutical Center, College of Pharmacy, University of Kentucky, 789 South Limestone Street, Lexington, KY 40536 USA; 2 Department of Pharmaceutical Sciences, College of Pharmacy, University of Kentucky, 789 South Limestone Street, Lexington, KY 40536 USA; 3 Department of Chemistry, University of Kentucky, 505 Rose Street, Lexington, KY 40506 USA; 4 Center for Pharmaceutical Research and Innovation, College of Pharmacy, University of Kentucky, 789 South Limestone Street, Lexington, KY 40536 USA

## Abstract

Human mPGES-1 is recognized as a promising target for next generation of anti-inflammatory drugs without the side effects of currently available anti-inflammatory drugs, and various inhibitors have been reported in the literature. However, none of the reported potent inhibitors of human mPGES-1 has shown to be also a potent inhibitor of mouse or rat mPGES-1, which prevents using the well-established mouse/rat models of inflammation-related diseases for preclinical studies. Hence, despite of extensive efforts to design and discover various human mPGES-1 inhibitors, the promise of mPGES-1 as a target for the next generation of anti-inflammatory drugs has never been demonstrated in any wild-type mouse/rat model using an mPGES-1 inhibitor. Here we report discovery of a novel type of selective mPGES-1 inhibitors potent for both human and mouse mPGES-1 enzymes through structure-based rational design. Based on *in vivo* studies using wild-type mice, the lead compound is indeed non-toxic, orally bioavailable, and more potent in decreasing the PGE_2_ (an inflammatory marker) levels compared to the currently available drug celecoxib. This is the first demonstration in wild-type mice that mPGES-1 is truly a promising target for the next generation of anti-inflammatory drugs.

## Introduction

As the principal pro-inflammatory prostanoid, prostaglandin E2 (PGE_2_) serves as a mediator of pain and fever in inflammatory reactions in a number of inflammation-related diseases^1^, such as chronic pains, cardiovascular diseases, neurodegenerative diseases, and cancers^2–4^. The biosynthesis^5^ of PGE_2_ starts from arachidonic acid (AA). Cyclooxygenase (COX)-1 or COX-2 converts AA to prostaglandin H2 (PGH_2_)^5^, and prostaglandin E synthase (PGES) transforms PGH_2_ to PGE_2_^6^. The first generation of nonsteroidal anti-inflammatory drugs (NSAIDs), such as aspirin used to treat pain and reduce fever or inflammation, inhibit both COX-1 and COX-2 without selectivity, and the second generation of NSAIDs, including celecoxib (Celebrex), rofecoxib (Vioxx) and valdecoxib (Bextra), selectively inhibit COX-2. The COX-2 specific inhibitors still have a number of serious side effects, such as increasing the risk of fatal heart attack or stroke and causing stomach or intestinal bleeding. The serious side effects led to withdrawal of rofecoxib and valdecoxib, although celecoxib still remains in clinical use. The serious side effects are due to the fact that the synthesis of all physiologically needed prostaglandins downstream of PGH_2_ are inhibited by the action of the COX-1/2 inhibitors. For example, blocking the production of prostaglandin-I_2_ (PGI_2_) will cause significant cardiovascular problems^7^.

Microsomal PGES-1 (mPGES-1), an inducible enzyme, is a more promising, ideal target for anti-inflammatory drugs, because the mPGES-1 inhibition will only block the PGE_2_ production without affecting the production of PGI_2_ and other prostaglandins, as confirmed by reported knock-out studies^8,9^. Specifically, the mPGES-1 expression in most tissues including heart and brain is low, but abundant in a limited number of organs including kidney^10,11^ and reproductive organs^12^. Protein mPGES-1 in human is related to various diseases associated with inflammation. For example, up-regulation of mPGES-1 was detected in heart tissue after myocardial infarction and in Alzheimer’s disease tissues^13,14^. Unlike the COX-1/2 inhibition, inhibition of terminal mPGES-1 will only block the production of PGE_2_ without affecting the normal production of other prostaglandins including PGI_2_. Reported knock-out studies identified mPGES-1 as an essential central switch in pyresis^8^. The mPGES-1 knock-out studies also revealed a decrease in inflammatory response in a collagen-induced arthritis model^9^. In contrast to COX-2, mPGES-1-deficient mice were reported to be viable, fertile and have normal phenotype^9^. Ischemic stroke induced in mPGES-1 null mice was reported to show significant reduction in the infarct size and volume^15,16^. Thus, mPGES-1 inhibitors are expected to retain the anti-inflammatory effect of COX-1/2 inhibitors, but without the side effects caused by the COX-1/2 inhibition. For development of a next generation of anti-inflammatory drugs, various mPGES-1 inhibitors have been reported in the literature^17–38^.

Unfortunately, none of the reported potent inhibitors of human mPGES-1 has shown to be also a potent inhibitor of mouse or rat mPGES-1, which prevents using the well-established mouse/rat models of inflammation-related diseases for preclinical studies. Here we report discovery of a novel type of mPGES-1 inhibitors potent for both human and mouse mPGES-1 enzymes through structure-based rational design. These inhibitors are also highly selective for mPGES-1 over COX-1/2 and orally bioavailable, enabling preclinical testing using the well-established wild-type mouse models of inflammation-related diseases through oral administration.

## Results

### Design and Synthesis of Dual Inhibitors of Human and Mouse mPGES-1 Proteins

**Figure 1:**
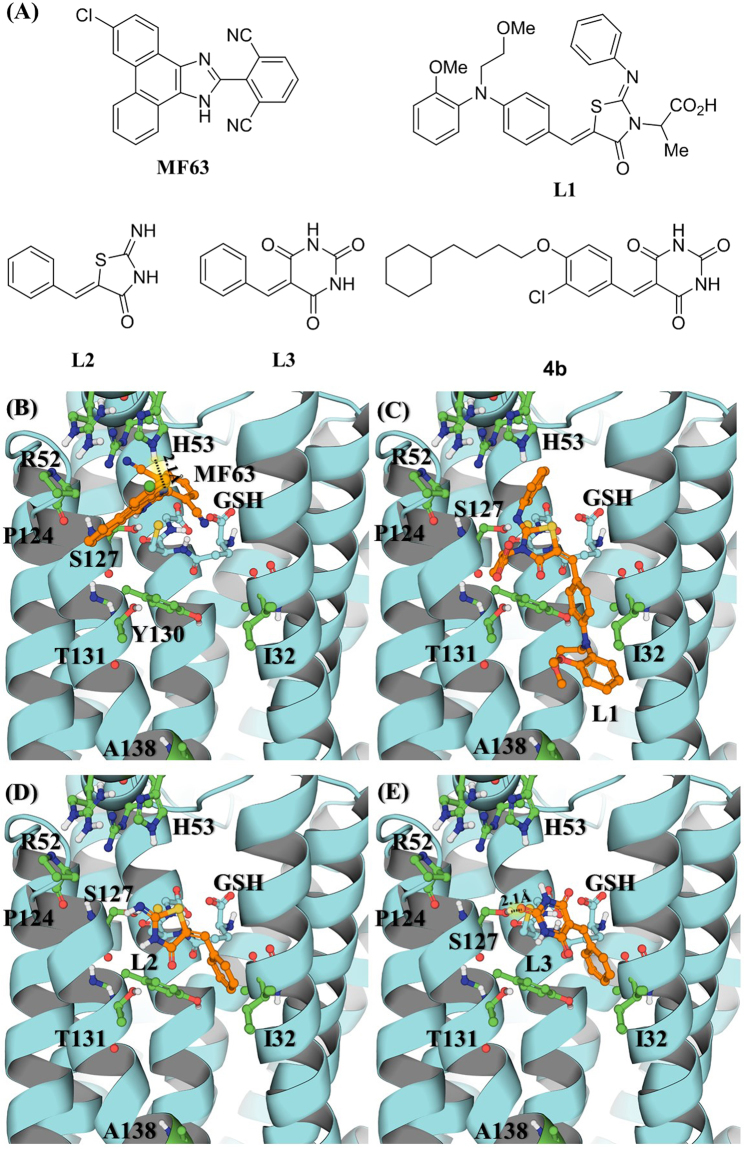
Molecular structures of ligands (MF63 and L1 to 3,) and their binding with human mPGES-1. (**A**) Ligand structures; (**B**) binding with MF63; (**C**) binding with L1; (**D**) binding with L2; (**E**) binding with L3.

Our rational design of novel mPGES-1 inhibitors started from molecular modeling of various human mPGES-1 inhibitors, including MF63^30^, L1^39^ and its scaffold structure (L2) depicted in Fig. 1A, for their binding with human and mouse mPGES-1 enzymes, and aimed to design a modified, novel compound which can favorably bind with both human and mouse mPGES-1 enzymes in the active site. To design a compound which can favorably bind with both human and mouse mPGES-1 enzymes, our strategy was to identify a scaffold structure which can bind in the conserved region of the active site, ensuring that the scaffold structure can bind with both of the enzymes in a similar binding mode. For this purpose, molecular docking was performed to understand the binding of known mPGES-1 inhibitors with both human and mouse mPGES-1 enzymes based on an X-ray crystal structure (PDB ID: 4BPM)^40^ of human mPGES-1 and a homology model of mouse mPGES-1 developed by using the human mPGES-1 structure as a template.

According to molecular docking, almost all of the known potent human mPGES-1 inhibitors (such as MF63 depicted in Fig. 1A and B) examined in our study have favorable interactions with unique residues (such as R52 which is K52 in mouse mPGES-1 and H53 which is R53 in mouse mPGES-1) of human mPGES-1, explaining why the known potent human mPGES-1 inhibitors cannot potently inhibit mouse mPGES-1. Nevertheless, as seen in Fig. 1C and D, L1 (identified from virtual screening)^39^, which has a unique scaffold structure (L2), binds in a conserved region of the active site in the human and mouse mPGES-1 enzymes, although L1 has a low binding affinity with human mPGES-1 (IC_50_ = 3.5 µM)^39^. The conserved region is nearby S127 and has a mainly hydrophobic pocket surrounded by Y28, I32, G35, L39, S127, Y130, T131, L135, and A138 for human mPGES-1. In comparison, mouse mPGES-1 differs from human mPGES-1 only in residues #32 (which is V32), #131 (which is V131), and #138 (which is F138). In Fig. 1B to E, we mainly highlight the residues of human mPGES-1 that are different in mouse mPGES-1, in addition to the most important residues (such as S127) for binding.

**Figure 2:**
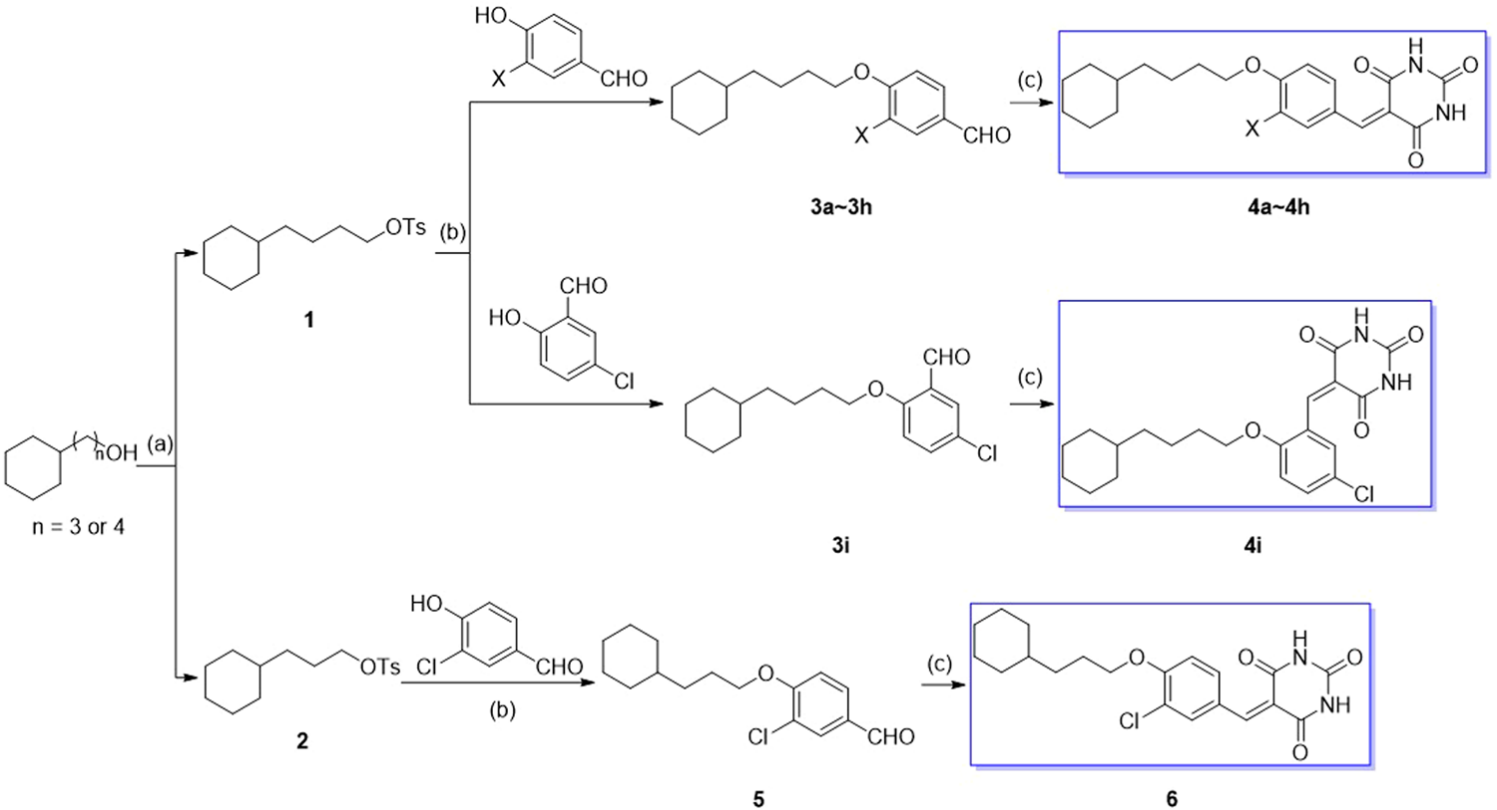
Synthetic protocol of benzylidenebarbituric acid derivatives 4a to 4i and 6. Reagent and conditions: (**a**) *p*-Toluenesulfonyl chloride (1.25 equiv.), 50% KOH aq., DCM, 0 °C~rt; (**b**) K_2_CO_3_ (2.0 equiv.), DMF, 80 °C; (**c**) Barbituric acid, EtOH/H_2_O (4:1, *v/v*), reflux.

On the basis of the scaffold structure L2, we designed a modified scaffold structure (L3, see Fig. 1A) which can favorably bind in the conserved region of the active site. Depicted in Fig. 1B to E are the binding structures of human mPGES-1 with MF63, L1, L2, and L3, respectively. As seen in Fig. 1D and E, compared to L2, L3 is a more favorable scaffold structure, as a carbonyl oxygen on the barbituric acid head group of L3 forms a hydrogen bond (HB) with the hydroxyl group of S127 side chain. The same HB is expected to exist in mouse mPGES-1 binding with L3. By using this novel scaffold (L3), a series of 10 potentially promising new compounds were designed, synthesized, characterized (see Supplementary Material for the structural characterization), and assayed for their *in vitro* inhibitory activities against human and mouse mPGES-1 enzymes. Depicted in Fig. 2 are the synthetic schemes, and summarized in Table 1 are the *in vitro* activity data.

**Table 1:** *In vitro* inhibitory potencies of synthesized benzylidenebarbituric acid derivatives.

Compound		IC_50_ (nM)^a^ against mPGES-1		COX-1/2 Inhibition (%) at 100 µM^b^
ID	X	Human mPGES-1	Mouse mPGES-1	
**4a**	H	622 ± 121	7080 ± 627	0 ± 15
**4b**	Cl	33 ± 3	157 ± 31	4.3 ± 6.0
**4c**	Br	45 ± 8	917 ± 321	63 ± 0.8
**4d**	Me	82 ± 10	18900 ± 4080	9.1 ± 6.6
**4e**	OH	116 ± 17	2900 ± 293	68 ± 3.4
**4f**	OMe	121 ± 20	146 ± 209	28 ± 3.6
**4g**	OEt	186 ± 26	2410 ± 339	0 ± 15.1
**4h**	NO_2_	67 ± 20	698 ± 97	43 ± 3.2
**4i**		87 ± 27	19100 ± 3490	56 ± 3.7
**6**		69 ± 16	292 ± 47	17 ± 4.6

^a^Data are expressed as the Mean ± SD of measurements in triplicate. ^b^The % inhibition of the compound at a concentration of 100 µM against the COX-1/2 (with equal amounts of COX-1 and COX-2 in terms of the enzyme activities).

### *In Vitro* Activities and Selectivity

**Figure 3:**
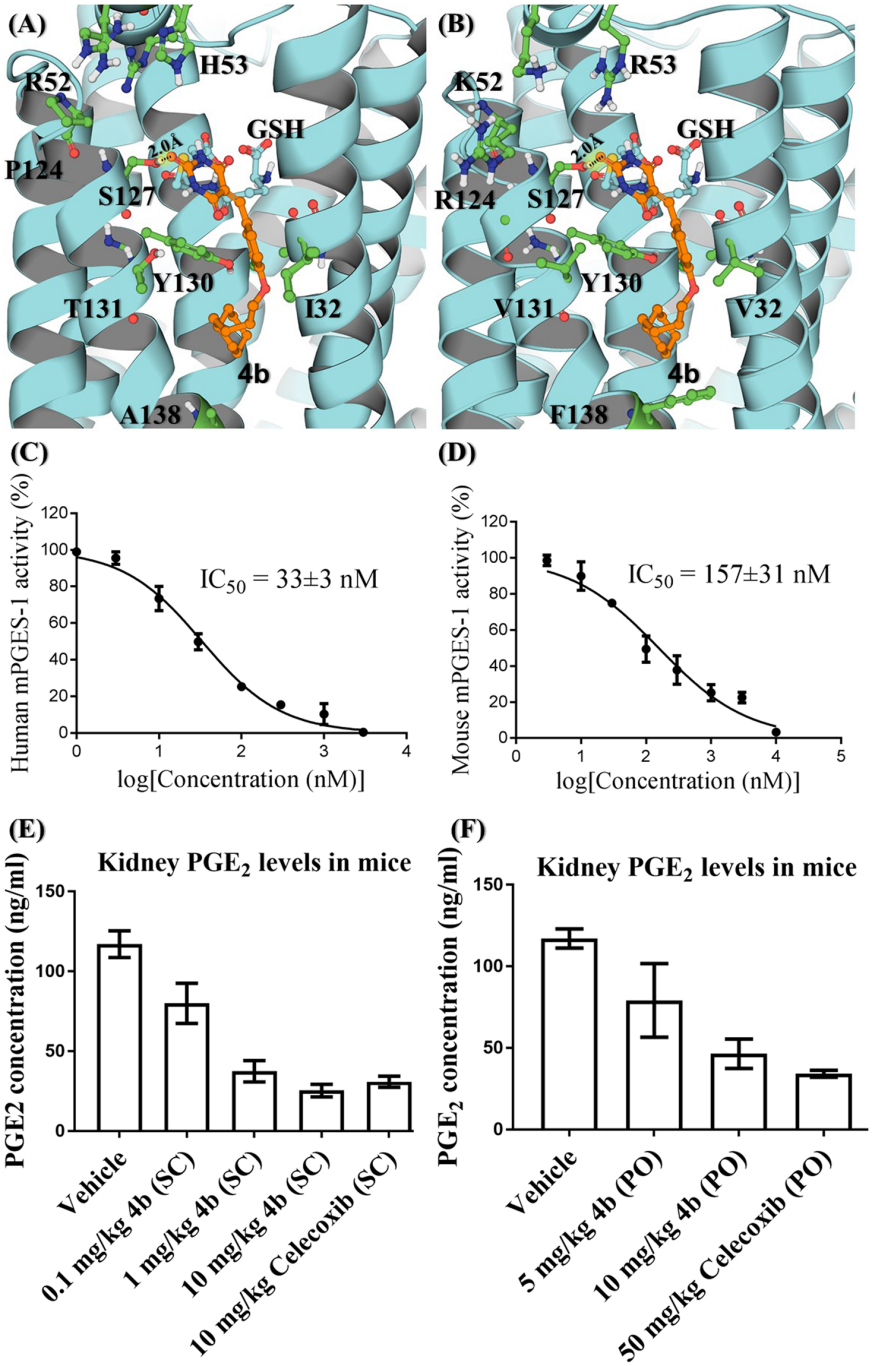
Compound **4b** binding with the enzymes and its *in vitro* and *in vivo* activities. (**A**) Binding with human mPGES-1; (**B**) binding with mouse mPGES-1; (**C**) dose-dependent inhibition of human mPGES-1 (n = 3); (**D**) dose-dependent inhibition of mouse mPGES-1 (n = 3); (**E**) and (**F**) data from *in vivo* assays using the mouse air-pouch model (n = 5 for each group) with **4b** or celecoxib given SC or PO. Normalized levels of PGE_2_ in kidney collected following the formation of air-pouches on the backs of mice and injection of the pro-inflammatory agent carrageenan to stimulate PGE2 synthesis. Mice were treated SC or PO with vehicle, **4b**, or celecoxib at various dose conditions for 24 hours prior to collection of the kidney samples (analyzed for PGE_2_ by ELISA). Statistical results from the one-way ANOVA analysis of the data in panel E with post hoc tests: *p* = 0.0035 for 0.1 mg/kg **4b** (SC) *vs* Vehicle; *p* < 0.0001 for 1 mg/kg **4b** (SC) *vs* Vehicle, 10 mg/kg **4b** (SC) *vs* Vehicle, and 10 mg/kg Celecoxib (SC) *vs* Vehicle; *p* = 0.0012 for 1 mg/kg **4b** (SC) *vs* 0.1 mg/kg **4b** (SC); *p* = 0.0002 for 10 mg/kg **4b** (SC) *vs* 0.1 mg/kg **4b** (SC); *p* = 0.0003 for 10 mg/kg Celecoxib (SC) *vs* 0.1 mg/kg **4b** (SC); *p* = 0.3176 for 10 mg/kg **4b** (SC) *vs* 1 mg/kg **4b** (SC); and *p* = 0.6424 for 10 mg/kg Celecoxib (SC) *vs* 10 mg/kg **4b** (SC). Statistical results from the one-way ANOVA analysis of the data in panel F with post hoc tests: *p* = 0.0281 for 5 mg/kg **4b** (PO) *vs* Vehicle; *p* = 0.0011 for 10 mg/kg **4b** (PO) *vs* Vehicle; *p* = 0.0008 for 50 mg/kg Celecoxib (PO) *vs* Vehicle; *p* = 0.0481 for 10 mg/kg **4b** (PO) *vs* 5 mg/kg **4b** (PO); *p* = 0.0221 for 50 mg/kg Celecoxib (PO) *vs* 5 mg/kg **4b** (PO); and *p* = 0.4986 for 50 mg/kg Celecoxib (PO) *vs* 10 mg/kg **4b** (PO).

Our protocols for the protein preparation and *in vitro* activity assays were the same as described in previous reports^39,41–43^. MK-886, a well-recognized human mPGES-1 inhibitor, was used as a reference compound for which we obtained IC_50_ = 2.6 ± 0.6 µM, which is close to the previously reported IC_50_ values (IC_50_ = 1.6 µM^33^, 2.4 ± 0.3 µM^19^) without significant inhibition of mouse mPGES-1. In addition, we obtained IC_50_ = 1.5 nM for MF63 against human mPGES-1 and MF63 at 10 µM had no significant inhibition against mouse mPGES-1, which is consistent with the previously reported data showing that MF63 potently inhibited human mPGES-1 (IC_50_ = 1.3 nM) without significant activity against the mouse or rat enzyme^30^. As seen in Table 1, the 10 compounds synthesized are all potent inhibitors of human mPGES-1 (IC_50_ = 33 to 620 nM). The most potent one is **4b** (IC_50_ = 33 nM). Based on the similarity of human and mouse mPGES-1 enzymes in this conserved region, these human mPGES-1 inhibitors were expected to similarly, but not equally, inhibit mouse mPGES-1. Indeed, these compounds can also significantly inhibit mouse mPGES-1, but with relatively lower potency (IC_50_ = 157 nM to ~19 µM). So, **4b** is the most potent inhibitor for both human and mouse mPGES-1 enzymes. Figure 3A and B depict the modeled structures of **4b** binding with human and mouse mPGES-1 enzymes, and Fig. 3C and D show the dose-response curves of **4b** against the enzymes. For the structure-activity relationship (SAR), we first investigated the impact of substitution on the central benzene ring on the inhibitory potency. It was observed that without a substituent, as for **4a** (X = H), the inhibitory potency of the compound against both human and mouse mPGES-1 enzymes was greatly impaired. Secondly, both electro-withdrawing and electro-donating groups were tolerated, although the former resulted in greater inhibition. The central benzene rings of **4i** was originated from salicylaldehydes with chlorine substituted on 5-position. **4i** was active against human mPGES-1 with submicromolar potency (IC_50_ = 87 ± 27 nM), but not effective against the mouse enzyme (IC_50_ > 10 µM). The length of the alkoxy side chain was also inspected in compounds **4b** and **6**. With one methylene group “cut off” from the side chain, **6** was less potent against both human and mouse mPGES-1 enzymes as compared to **4b**.

Next we wanted to know whether **4b** and the other compounds listed in Table 1 have significant inhibitory activities against either COX-1 or COX-2. For this purpose, these compounds were assayed for their potential inhibitory activities against mixed COX-1 and COX-2 (denoted as COX-1/2) with equal amounts of COX-1 and COX-2 in terms of the enzyme activities. As seen in Table 1, only three compounds (**4c**, **4e**, and **4i**) at a concentration of 100 µM inhibited COX-1/2 greater than 50%. Three other compounds (**4f**, **4h**, and **6**) at a concentration of 100 µM did significantly inhibit COX-1/2, but the inhibition was less than 50%. Notably, the most potent compound **4b** and three other compounds (**4a**, **4d**, and **4 g**) at a concentration of 100 µM did not significantly inhibit COX-1/2. These four compounds are highly selective for the mPGES-1 enzymes over COX-1/2. So, **4b** was identified as our lead compound.

### *In Vivo* Anti-inflammatory Activity

To examine the anti-inflammatory potential of **4b**, we determined the *in vivo* effectiveness of **4b** in the most popularly used mouse air-pouch model of inflammation in comparison with celecoxib. The air-pouch model of inflammation^30,44^ is widely used for determining the *in vivo* effectiveness of inhibitors of prostaglandin synthesis. Air pouches were produced by duplicate injections of 3 mL of sterile air under the skin on the back of mice. After the formation of the air-pouch, a single injection of the inflammatory agent carrageenan into the pouch resulted in the recruitment of inflammatory cells and the production of a fluid exudate containing significant levels of PGE_2_ (an inflammatory marker) produced primarily by activities of COX-2 and mPGES-1. Then, the mice were treated SC (subcutaneous) or PO (oral gavage) with a single dose of **4b**, celecoxib, or vehicle for 24 hours prior to collection of air-pouch fluid and the kidney samples. The air-pouch fluid and kidney samples were analyzed for PGE_2_ by the same ELISA method used in the *in vitro* enzyme activity assay mentioned above. As mPGES-1 is more abundant in kidney, we examined the effects of **4b** and celecoxib on the PGE_2_ level in kidney. Depicted in Fig. 3E and F are the measured PGE_2_ levels in kidney; the corresponding data in air-pouch fluid (not shown) are similar.

**Figure 4:**
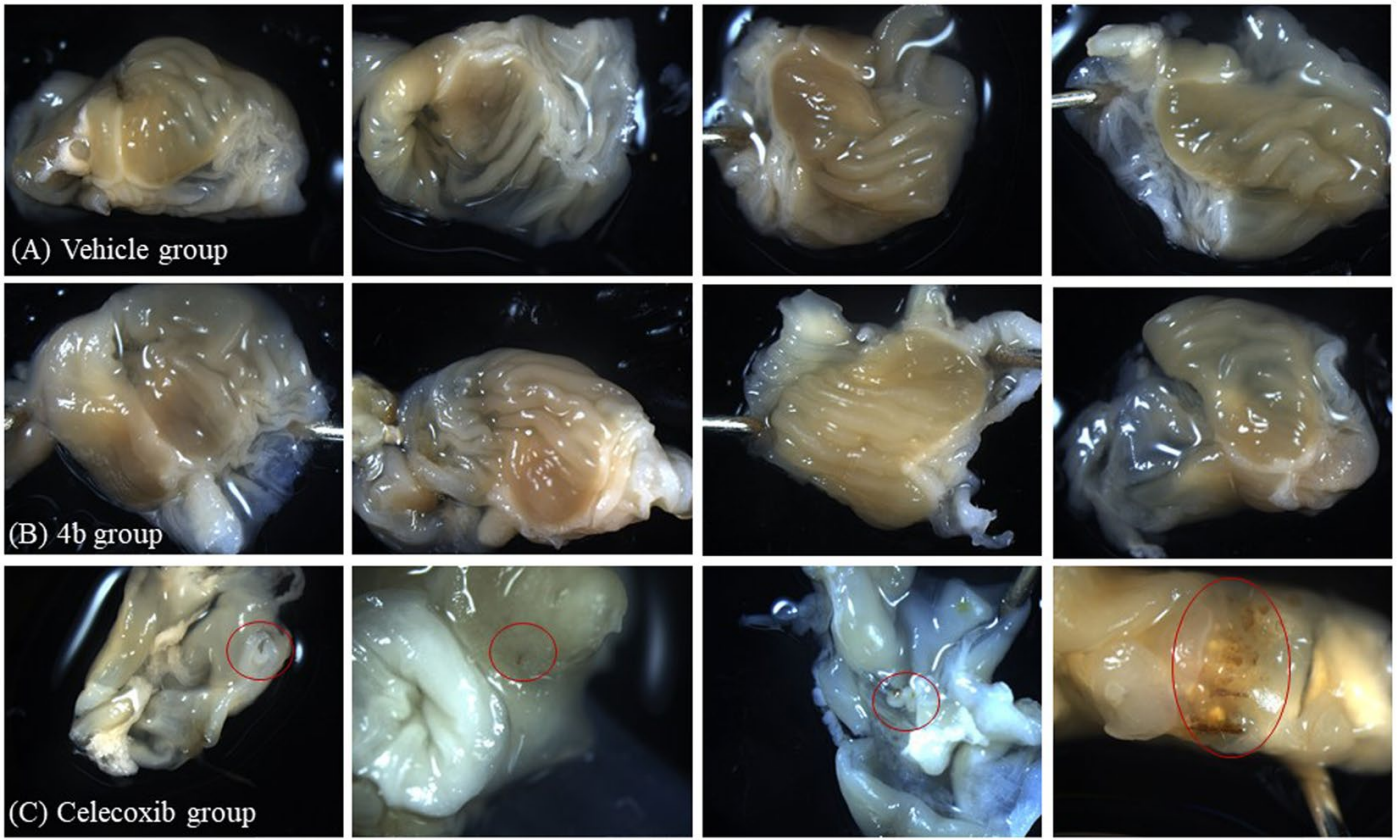
Representative images of stomach tissues collected from mice at 24 hr after PO administration of (**A**) vehicle (oil) or (**B**) **4b** (1 g/kg in oil) or (**C**) celecoxib (50 mg/kg in oil). For all mice in the vehicle and **4b** groups, we did not find any bleeding spot on the inner side of stomach samples. Meanwhile, for each mouse in the celecoxib group, we were able to clearly see at least one bleeding spot; the bleeding points are labeled in red circles.

As shown in Fig. 3E and **4b** administered SC at each dose (0.1, 1, or 10 mg/kg) condition significantly decreased the PGE_2_ levels in mice (*p* = 0.0035, *p* < 0.0001, and *p* < 0.0001, respectively). Even the low dose (0.1 mg/kg) of **4b** administered SC significantly decreased the PGE_2_ levels in mice (Fig. 3E). Compared to the dose of 0.1 mg/kg, 1 mg/kg **4b** was significantly more potent (*p* = 0.0012). However, further increase of the dose from 1 mg/kg to 10 mg/kg did not significantly improve the *in vivo* potency (*p* = 0.3176), which may be interpreted as the possibility that 1 mg/kg **4b** (SC) has nearly reached the ceiling for decreasing the PGE_2_ level through the complete mPGES-1 inhibition. In addition, there was also no significant difference in the *in vivo* potency between 1 mg/kg **4b** and 10 mg/kg celecoxib and between 10 mg/kg **4b** and 10 mg/kg celecoxib.

Further, according to Fig. 3F and 4b administered PO at each dose (5 or 10 mg/kg) condition also significantly decreased the PGE_2_ levels in mice (*p* = 0.0281 and *p* = 0.0011, respectively) in a dose-dependent manner (*p* = 0.0481), showing the feasibility of oral administration for practical therapeutic applications. There was no significant difference in the *in vivo* potency between the 10 mg/kg **4b** and 50 mg/kg celecoxib (*p* = 0.4986).

Finally, we also tested acute toxicity/safety of **4b** in comparison with celecoxib. In fact, 50 mg/kg celecoxib administered PO were very toxic for stomach and other issues of mice, and bleeding ulcer was observed at gastric mucosa. In comparison, a high dose (up to 1 g/kg) of **4b** administered PO did not cause any toxic sign in mice during our observation for 14 days. Depicted in Fig. 4 are representative images of the stomach tissues collected from mice at 24 hr after the PO administration of vehicle or **4b** or celecoxib.

## Discussion

As well known, inflammation is related to many types of diseases, and mPGES-1 was recognized as the most promising target for developing the highly desirable next generation of anti-inflammatory drugs without the adverse side effects of currently used COX inhibitors. The promise of mPGES-1 as the target was based on understanding of the physiological process and biosynthesis of the pro-inflammatory compound PGE_2_, and was supported strongly by the mPGES-1 gene knock-out studies^8,9^. It is well known that mouse/rat models of inflammation-related diseases have been well-established, enabling to test a potentially promising anti-inflammatory drug candidate in the established mouse/rat models of inflammation-related diseases. Unfortunately, despite of extensive efforts to design and discover various human mPGES-1 inhibitors and the fact that numerous potent inhibitors of human mPGES-1 have already been reported in the literature, the promise of mPGES-1 as a target for the next generation of anti-inflammatory drugs has never been demonstrated in any wild-type mouse/rat model using an inhibitor of mPGES-1 because none of the previously discovered human mPGES-1 inhibitors can potently inhibit mouse/rat mPGES-1. Without a dual inhibitor (against both human and mouse mPGES-1 enzymes) available, one has to explore alternative animal models by using either other animal species that are less popular for use as animal models of inflammation-related diseases or mPGES-1 gene knock-out/knock-in mice expressing human mPGES-1 instead of mouse mPGES-1. For example, Merck developed the first strain of mPGES-1 gene knock-out/knock-in mice expressing human mPGES-1 instead of mouse mPGES-1^30^. But interpretation of the animal data with the knock-in mice is complicated due to the difference between the original mouse gene and knock-in gene in the localization and amount. So, there is still no clinically useful mPGES-1 inhibitor developed so far. Reported here is the first demonstration that a potent human mPGES-1 inhibitor has potent *in vivo* activity in wild-type mice-based air-pouch model of inflammation. So, we are able to demonstrate in wild-type mice that mPGES-1 is truly a promising target for the next generation of anti-inflammatory drugs.

Compared to the currently used COX-2 inhibitor (celecoxib) in clinic, **4b** is not only more potent in decreasing the PGE_2_ levels, but also much safer. In addition, **4b** is orally bioavailable, which is crucially important for development of an oral drug as it is usually very difficult to achieve the highly desirable oral bioavailability in drug development. For all of these reasons, **4b** may be developed for the truly promising next-generation anti-inflammatory drug for oral administration without the side effects of the currently available anti-inflammatory drugs.

In general, a traditional drug discovery and development effort is usually focused on identification of ligands of a human protein target without accounting for the species difference in target protein during the early drug design and discovery stage before finding out that the ligands identified *in vitro* are actually inactive in the *in vivo* animal models. Our study demonstrates a more effective strategy of drug design and discovery to rationally design a dual inhibitor of human and animal target proteins. The general strategy of our structure-based rational design of a dual inhibitor of the human and mouse mPGES-1 enzymes may also be used for other drug targets with significant species differences in the binding pocket.

## Materials and Methods

### General

All the final products described here were verified to have a purity of 95% or higher as determined by HPLC. The typical synthetic methods of **4b** are shown below. All other information including the computational studies, synthesis and characterization data of intermediates and final products, *in vitro* and *in vivo* experiments, and NMR spectra are described in supporting information. GraphPad Prism 7 software (GraphPad Software, La Jolla, CA) was used to perform the one-way analysis of variance (ANOVA) with post hoc tests, allowing us to examine the signifcance of the difference in the *in vivo* activity data between each pair of dose conditions. *p* < 0.05 was considered statistically significant.

### Materials

Reference compounds MK-886 and MF63 were purchased from Cayman Chemical (Ann Arbor, MI). All starting chemicals for our synthesis were purchased from Sigma-Aldrich (St. Louis, MO) or Fisher Scientific (Hampton, NH) and used without further purification. Compounds were purified by SiO_2_ flash chromatography (Flash silica gel 32–63 u, Dynamic Adsorbents Inc., Norcross, GA). ^1^H and ^13^C NMR spectra were recorded on a Varian Unity Inova 400 MHz spectrometer (Palo Alto, CA) at ambient temperature using 99.8% CDCl_3_ and 99.9% DMSO-d6 (Cambridge Isotope Laboratories, Tewksbury, MA). ^1^H and ^13^C chemical shifts were referenced to internal solvent resonances and reported in parts per million (ppm), with coupling constants *J* given in Hz. HR-ESI-MS spectra were recorded on AB SCIEX Triple TOF 5600 system (AB Sciex, Framingham, MA). Purity was determined by HPLC (Waters 1525 Binary HPLC pump, Waters 2487 Dual λ Absorbance Detector, and Waters 717plus Autosampler, Milford, MA) at λ of 370 nm using acetonitrile and 0.1% TFA (70:30) as mobile phase. All of the final products described here were verified to have a purity of 95% or higher. Wesson vegetable oil (Memphis, TN) was used as vehicle in the *in vivo* experiments.

Wild-type CD-1 mice (28–35 g) were ordered from Harlan (Indianapolis, IN), and housed for a week prior to the experimental studies. All animals were allowed ad libitum access to food and water and maintained on a 12 h light/12 h dark cycle, with the lights on at 8:00 am at a room temperature of 21–22 °C. Experiments were performed in a same colony room in accordance with the Guide for the Care and Use of Laboratory Animals as adopted and promulgated by the National Institutes of Health. The animal protocol was approved by the IACUC (Institutional Animal Care and Use Committee) at the University of Kentucky.

### Preparation of 1

Aqueous KOH solution (50%, 20 mL) was added to the solution of 4-cyclohexyl-1-butanol (1.05 g, 6.72 mmol, 1.00 equiv.) in dichloromethane (40 mL). The mixture was brought to 0~5 °C using ice-bath, and *p*-toluenesulfonyl chloride (1.54 g, 8.06 mmol, 1.20 equiv.) was added portionwise over a period of 30 min. The resulting reaction mixture was stirred at room temperature for 5 h and partitioned between CH_2_Cl_2_ (30 mL) and water (30 mL). The organic layer was isolated and the aqueous layer was extracted with CH_2_Cl_2_ (30 mL × 3). The combined organic phase was washed sequentially with water (30 mL) saturated NaHCO_3_ solution (30 mL) and brine (30 mL), dried over anhydrous Na_2_SO_4_, and evaporated under reduced pressure^45^. The residue was dried under high vacuum using oil pump overnight to afford the tosylate **1** as white wax in high purity (Yield: 1.98 g, 95%).

### Preparation of 3b

The suspension of 3-chloro-4-hydroxybenzaldehyde (0.32 g, 2.04 mmol, 1.00 equiv.), 4-cyclohexyl-1-butanol tosylate (**1**) (0.63 g, 2.04 mmol, 1.00 equiv.) and potassium carbonate (0.56 g, 4.09 mmol, 2.00 equiv.) in DMF (10 mL) was heated at 80 °C for 12 h (or overnight). The reaction mixture was then diluted with water (20 mL) and extracted with ethyl acetate (30 mL × 3). The combined organic phase was washed sequentially with saturated NaHCO_3_ solution (30 mL), water (30 mL) and brine (30 mL), dried over anhydrous Na_2_SO_4_, evaporated under reduced pressure and dried under vacuum at room temperature^46^. The crude product was used in subsequent step without further purification. However, the analytical sample can be obtained as light yellow oil by flash chromatography on silica gel using a mixture of hexanes and EtOAc (4:1) as eluent (Yield: 0.53 g, 88%).

### Preparation of 4b

The suspension of **3b** (0.30 g, 1.02 mmol, 1.00 equiv.) and barbituric acid (0.13 g, 1.02 mmol, 1.00 equiv.) in absolute ethanol and distilled water (4:1, *v/v*) was heated at reflux for 5 h and the reaction mixture was cooled to room temperature. The precipitate was filtered off, washed with hot water and ethanol, and dried under vacuum to afford the product as yellow powders in high purity (0.37 g, 90%). The analytical sample was obtained by recrystallization from a mixture of ethanol and DMF^47,48^. ^1^H NMR (400 MHz, DMSO) δ 11.34 (s, 1 H), 11.23 (s, 1 H), 8.66 (d, *J* = 2.1 Hz, 1 H), 8.20 (s, 1 H), 8.15 (dd, *J* = 9.1, 2.2 Hz, 1 H), 7.25 (d, *J* = 8.9 Hz, 1 H), 4.18 (t, *J* = 6.4 Hz, 2 H), 1.82–1.49 (m, 7 H), 1.49–1.36 (m, 2 H), 1.30–1.02 (m, 6 H), 0.97–0.73 (m, 2 H). ^13^C NMR (101 MHz, DMSO) δ 163.58, 162.12, 157.54, 153.26, 150.13, 136.54, 135.20, 125.66, 120.96, 116.92, 112.94, 69.09, 36.94, 36.41, 32.80, 28.57, 26.20, 25.83, 22.59. HRMS (ESI+) *m/z* calcd for C_21_H_26_ClN_2_O_4_ (MH)^+^: 405.1576, found: 405.1574. HPLC purity: 98.0%; t_R_ = 14.436.

## Electronic supplementary material

Supplementary Materials
