# Supplementary material for: Structure-based discovery of mPGES-1 inhibitors suitable for preclinical testing in wild-type mice as a new generation of anti-inflammatory drugs
Source: Sci Rep. 2018 Mar 26;8:5205. doi: 10.1038/s41598-018-23482-4 (PMC5979965; doi:10.1038/s41598-018-23482-4)
Supplement: Supplementary file 1 — Supplementary Materials [file 41598_2018_23482_MOESM1_ESM.pdf]

Supporting Information

Structure-based discovery of mPGES-1 inhibitors suitable for preclinical testing in wild-type mice as a new generation of anti-inflammatory drugs

Kai Ding,[1,2,3,†] Ziyuan Zhou,[1,2,†] Shurong Hou,[2] Yaxia Yuan,[1,2,4] Shuo Zhou,[1,2] Xirong Zheng,[2] Jianzhong Chen,[1,2] Charles Loftin,[2] Fang Zheng,[1,2] and Chang-Guo Zhan*[,1,2,4]

[1]Molecular Modeling and Biopharmaceutical Center, College of Pharmacy, University of Kentucky, 789 South Limestone Street, Lexington, KY 40536. [2]Department of Pharmaceutical Sciences, College of Pharmacy, University of Kentucky, 789 South Limestone Street, Lexington, KY 40536. [3]Department of Chemistry, University of Kentucky, 505 Rose Street, Lexington, KY 40506. [4]Center for Pharmaceutical Research and Innovation, College of Pharmacy, University of Kentucky, 789 South Limestone Street, Lexington, KY 40536.

[†] These authors contributed equally to this work.

Table of contents:

1. Computational studies **S2**
Trimer structure of human mPGES-1 **S2**
Trimer structure of mouse mPGES-1 **S2**
Molecular docking **S2**
2. Chemistry **S3**
General **S3**
Preparation of the tosylates (**1** and **2**) **S3**
Preparation of the aldehyde intermediates **3a**~**3i** and **5** **S4**
Preparation of the benzylidenenbarbituric acid derivatives **4a**~**4i** and **6** **S7**
Purity data of final products (**4a**~**4i** and **6**) as determined by HPLC **S12**
3. *In vitro* experimental tests **S12**
Preparation of mPGES-1 enzymes **S12**
Activity assays using a recombinant mPGES-1 **S13**
Activity assays of COX-1/2 **S13**
4. *In vivo* experiments **S13**
5. ^{1}H and ^{13}C NMR spectra of products 4a~4i and 6 **S15**
6. References **S25**

Materials and Methods

1. Computational studies

Trimer structure of human mPGES-1. The trimer structure of human mPGES-1 was generated by the symmetry operation *via* **PyMol**,[1] based on the X-ray crystal structure of the monomer unit for human mPGES-1(PDB ID: 4BPM).[2,3]

Trimer structure of mouse mPGES-1. As there is no available X-ray crystal structure of mouse mPGES-1, homology modeling was performed to model the structure of mouse mPGES-1. Sequence alignment was performed on the amino acid sequence of human mPGES-1 (accession number of O14684) and mouse mPGES-1 (accession number of Q9JM51) by using the Basic Local Alignment Search Tool (BLAST) of Uniprot server (http://www.uniprot.org/align/)[4,5]. The aligned sequence indicates that human mPGES-1 is highly homologous to mouse mPGES-1, with the sequence identity being 79% and the sequence similarity being 84%. As 40% sequence identity between a template protein and a target protein is considered to be sufficient for constructing a satisfactory homology model,[6,7] we built the trimer structure of mouse mPGES-1 based on the aforementioned trimer structure of human mPGES-1 by using the Modeller module[6] of **Discovery Studio 2.5.5**.[8] The GSH cofactor in human mPGES-1 structure was directly transferred to the mouse mPGES-1 model. The final structure was selected from 50 candidate models according to the DOPE score.[9]

Molecular docking. In order to explore the binding mode of the presented compounds with human and mouse mPGES-1, molecular docking was performed by using the program of **AutoDock 4.2**[10]. During the process of molecular docking, Lamarckian genetic algorithm was applied to treat the intermolecular interactions between ligand and protein. The number of the runs was set to 200, and the number of individuals for each run was set to 300. The maximum number of energy evaluations was set to 25,000,000, and the maximum number of generations to 300,000. The size of grid box was 80 × 80 × 80 points along each axis and the grid spacing was 0.2 Å. All of the candidates generated from the docking operations were evaluated and ranked in terms of binding free energies by using the standard energy score function implemented in the **AutoDock 4.2** program. The largest one of the docked binding structure clusters was selected as the finally chosen initial mPGES-1-ligand complex structure, based on the lowest binding free energy (*i.e.* the best energy score from the **AutoDock 4.2** scoring function) along with good geometric matching quality with the surrounding residues in the binding site of mPGES-1.

2. Chemistry

General. All the starting chemicals were purchased from Sigma-Aldrich (St. Louis, MO) or Fisher Scientific (Hampton, NH) and used without further purification. Compounds were purified by SiO_2 flash chromatography (Flash silica gel 32-63 u, Dynamic Adsorbents Inc., Norcross, GA). ^1H and ^{13}C NMR spectra were recorded on a Varian Unity Inova 400 MHz spectrometer (Palo Alto, CA) at ambient

temperature using 99.8% $CDCl_3$ and 99.9% DMSO-d6 (Cambridge Isotope Laboratories, Tewksbury, MA). ^{1}H and ^{13}C chemical shifts were referenced to internal solvent resonances and reported in parts per million (ppm), with coupling constants *J* given in Hz. HR-ESI-MS spectra were recorded on AB SCIEX Triple TOF 5600 system (AB Sciex, Framingham, MA). Purity was determined by HPLC (Waters 1525 Binary HPLC pump, Waters 2487 Dual λ Absorbance Detector, and Waters 717plus Autosampler, Milford, MA) at λ of 370 nm using acetonitrile and 0.1% TFA (70:30) as mobile phase. All the final products described here were verified to have a purity of 95% or higher.

Preparation of the tosylates (1 and 2)

Preparation of 1.[11] To the solution of 4-cyclohexyl-1-butanol (1.05 g, 6.72 mmol, 1.00 equiv.) in dichloromethane (40 mL) was added aqueous KOH solution (50 %, 20 mL). The mixture was brought to 0~5 ^{o}C using ice-bath and *p*-toluenesulfonyl chloride (1.54 g, 8.06 mmol, 1.20 equiv.) was added portionwise over a period of 30 min. The resulting reaction mixture was stirred at room temperature for 5 h and partitioned between CH_2Cl_2 (30 mL) and water (30 mL). The organic layer was isolated and the aqueous layer was extracted with CH_2Cl_2 (30 mL×3). The combined organic phase was washed sequentially with water (30 mL) saturated $NaHCO_3$ solution (30 mL) and brine (30 mL), dried over anhydrous Na_2SO_4, and evaporated under reduced pressure. The residue was dried under high vacuum using oil pump overnight to afford the tosylate **1** as white wax in high purity (Yield: 1.98 g, 95 %). The tosylation (synthesis of **2**) of 3-cyclohexyl-1-propanol followed the same protocol as described.

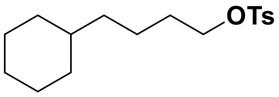

4-cyclohexylbutyl 4-methylbenzenesulfonate (1) Yield: 95 %. White wax-like solid. ^{1}H NMR (400 MHz, $CDCl_3$) δ 7.79 (d, *J* = 8.2 Hz, 2H), 7.34 (d, *J* = 6.3 Hz, 2H), 4.02 (t, 2H), 2.45 (d, *J* = 2.0 Hz, 3H), 1.71 – 1.58 (m, 7H), 1.58 – 0.82 (m, 14H), 0.82 – 0.36 (m, 3H). ^{13}C NMR (101 MHz, $CDCl_3$) δ 144.75, 133.36, 129.93, 128.03, 70.87, 37.57, 36.82, 33.39, 29.23, 26.79, 26.48, 22.76, 21.78.

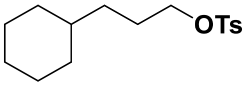

3-Cyclohexylpropyl 4-methylbenzenesulfonate (2) Yield: 67 %. Colorless oil. ^{1}H NMR (400 MHz, $CDCl_3$) δ 7.79 (d, *J* = 8.3 Hz, 2H), 7.35 (d, *J* = 8.0 Hz, 2H), 4.00 (t, *J* = 6.6 Hz, 2H), 2.44 (s, 3H), 1.71 – 1.55 (m, 7H), 1.25 – 1.04 (m, 6H), 0.88 – 0.71 (m, 2H). ^{13}C NMR (101 MHz, $CDCl_3$) δ 144.67, 133.12, 129.80, 127.85, 71.07, 36.96, 33.08, 32.83, 26.52, 26.21, 26.17, 21.61.

Preparation of the aldehyde intermediates 3a~3i and 5

Preparation of 3b.[12] The suspension of 3-chloro-4-hydroxybenzaldehyde (0.32 g, 2.04 mmol, 1.00 equiv.), 4-cyclohexyl-1-butanol tosylate (**1**) (0.63 g, 2.04 mmol, 1.00 equiv.) and potassium carbonate (0.56 g, 4.09 mmol, 2.00 equiv.) in DMF (10 mL) was heated at 80 ^{o}C for 12 h (or overnight). The reaction mixture was then diluted with water (20 mL) and extracted with ethyl acetate (30 mL×3). The combined

organic phase was washed sequentially with saturated $NaHCO_3$ solution (30 mL), water (30 mL) and brine (30 mL), dried over anhydrous Na_2SO_4, evaporated under reduced pressure and dried under vacuum at room temperature. The crude product was used in subsequent step without further purification. However, the analytical sample can be obtained as light yellow oil by flash chromatography on silica gel using a mixture of hexanes and EtOAc (4:1) as eluent. The preparation of other aldehyde intermediates (**3a**, **3c**~**3i**, and **5**) followed the similar protocol as described, with different starting materials.

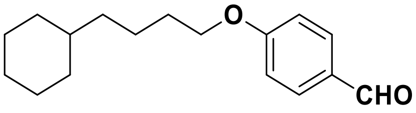

4-(4-Cyclohexylbutoxy)benzaldehyde (3a) Obtained from the reaction of 4-hydroxybenzaldehyde and **1**. Yield: 76 %. Colorless oil. ^{1}H NMR (400 MHz, $CDCl_3$) δ 9.86 (s, 1H), 7.80 (d, *J* = 8.7 Hz, 2H), 6.97 (d, *J* = 8.6 Hz, 2H), 4.02 (t, *J* = 6.5 Hz, 2H), 1.81 – 1.60 (m, 7H), 1.50 – 1.41 (m, 2H), 1.26 – 1.07 (m, 6H), 0.92 – 0.81 (m, 2H). ^{13}C NMR (101 MHz, $CDCl_3$) δ 191.14, 164.67, 132.36, 130.15, 115.15, 68.84, 37.99, 37.54, 33.77, 29.76, 27.11, 26.81, 23.65.

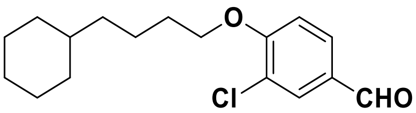

3-Chloro-4-(4-cyclohexylbutoxy)benzaldehyde (3b) Obtained from the reaction of 3-chloro-4-hydroxybenzaldehyde and **1**. Yield: 88 %. Light yellow oil. ^{1}H NMR (400 MHz, $CDCl_3$) δ 9.82 (s, 1H), 7.87 (d, *J* = 2.0 Hz, 1H), 7.72 (dd, *J* = 8.5, 2.0 Hz, 1H), 6.99 (d, *J* = 8.5 Hz, 1H), 4.09 (t, *J* = 6.5 Hz, 2H), 1.87 – 1.79 (m, 2H), 1.73 – 1.59 (m, 5H), 1.54 – 1.45 (m, 2H), 1.28 – 1.07 (m, 6H), 0.92 – 0.80 (m, 2H). ^{13}C NMR (101 MHz, $CDCl_3$) δ 189.75, 159.58, 131.26, 130.52, 130.08, 124.02, 112.55, 69.61, 37.60, 37.13, 33.43, 29.24, 26.79, 26.48, 23.22.

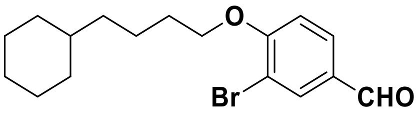

3-Bromo-4-(4-cyclohexylbutoxy)benzaldehyde (3c) Obtained from the reaction of 3-bromo-4-hydroxybenzaldehyde and **1**. Yield: 86%. Yellow oil. ^{1}H NMR (400 MHz, $CDCl_3$) δ 9.82 (s, 1H), 8.06 (d, *J* = 1.9 Hz, 1H), 7.78 (dd, *J* = 8.5, 1.8 Hz, 1H), 6.96 (d, *J* = 8.5 Hz, 1H), 4.10 (t, *J* = 6.4 Hz, 2H), 1.89 – 1.79 (m, 2H), 1.74 – 1.60 (m, 5H), 1.56 – 1.46 (m, 2H), 1.28 – 1.04 (m, 6H), 0.95 – 0.80 (m, 2H). ^{13}C NMR (101 MHz, $CDCl_3$) δ 189.69, 160.41, 134.67, 131.18, 130.54, 113.08, 112.40, 69.70, 37.61, 37.15, 33.45, 29.24, 26.81, 26.51, 23.26.

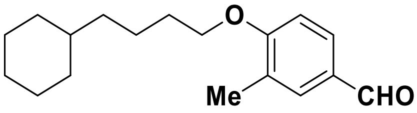

4-(4-Cyclohexylbutoxy)-3-methylbenzaldehyde (3d) Obtained from the reaction of 4-hydroxy-3-methylbenzaldehyde and **1**. Yield: 82 %. Orange oil. ^{1}H NMR (400 MHz, $CDCl_3$) δ 9.84 (s, 1H), 7.68 (d, *J* = 7.3 Hz, 2H), 6.89 (d, *J* = 7.7 Hz, 1H), 4.04 (t, *J* = 5.5 Hz, 2H), 2.26 (s, 3H), 1.86 – 1.77 (m, 2H), 1.74 – 1.62 (m, 5H), 1.54 – 1.44 (m, 2H), 1.28 – 1.11 (m, 6H), 0.93 –

0.80 (m, 2H). ^{13}C NMR (101 MHz, $CDCl_3$) δ 191.31, 162.64, 131.57, 130.79, 129.31, 127.83, 110.48, 68.46, 37.70, 37.26, 33.49, 29.50, 26.84, 26.54, 23.43, 16.38.

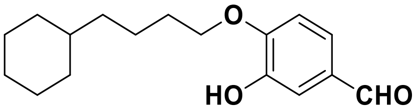

4-(4-Cyclohexylbutoxy)-3-hydroxybenzaldehyde (3e) Obtained from the reaction of 3,4-dihydroxybenzaldehyde and **1**. Yield: 74 %. White solid. ^{1}H NMR (400 MHz, $CDCl_3$) δ 9.82 (s, 1H), 7.47 – 7.36 (m, 2H), 6.94 (d, *J* = 8.2 Hz, 1H), 4.12 (t, *J* = 6.6 Hz, 2H), 1.87 – 1.77 (m, 2H), 1.73 – 1.61 (m, 5H), 1.51 – 1.41 (m, 2H), 1.26 – 1.11 (m, 6H), 0.93 – 0.83 (m, 2H). ^{13}C NMR (101 MHz, $CDCl_3$) δ 191.14, 151.45, 146.32, 130.55, 124.62, 114.17, 111.02, 69.44, 37.68, 37.21, 33.46, 29.40, 26.78, 26.49, 23.32.

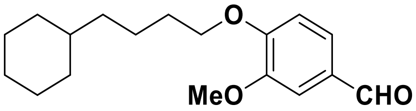

4-(4-Cyclohexylbutoxy)-3-methoxybenzaldehyde (3f) Obtained from the reaction of vanillin and **1**. Yield: 78 %. Colorless oil. ^{1}H NMR (400 MHz, $CDCl_3$) δ 9.82 (s, 1H), 7.44 – 7.35 (m, 2H), 6.94 (d, *J* = 8.1 Hz, 1H), 4.07 (t, *J* = 6.8 Hz, 2H), 3.90 (s, 3H), 1.88 – 1.79 (m, 2H), 1.71 – 1.58 (m, 5H), 1.50 – 1.40 (m, 2H), 1.25 – 1.05 (m, 6H), 0.91 – 0.80 (m, 2H). ^{13}C NMR (101 MHz, $CDCl_3$) δ 190.92, 154.29, 149.93, 129.94, 126.83, 111.48, 109.38, 69.28, 56.09, 37.59, 37.18, 33.42, 29.27, 26.77, 26.47, 23.24.

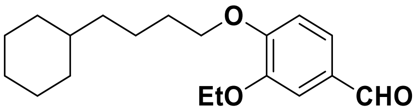

4-(4-Cyclohexylbutoxy)-3-ethoxybenzaldehyde (3g) Obtained from the reaction of 3-ethoxy-4-hydroxybenzaldehyde and **1**. Yield: 76 %. Colorless oil. ^{1}H NMR (400 MHz, $CDCl_3$) δ 9.83 (s, 1H), 7.45 – 7.34 (m, 2H), 6.95 (d, *J* = 6.1 Hz, 1H), 4.19 – 4.02 (m, 4H), 1.89 – 1.80 (m, 2H), 1.76 – 1.60 (m, 5H), 1.52 – 1.42 (m, 5H), 1.28 – 1.10 (m, 6H), 0.93 – 0.84 (m, 2H). ^{13}C NMR (101 MHz, $CDCl_3$) δ 191.06, 154.68, 149.29, 129.98, 126.73, 111.85, 111.04, 69.30, 64.69, 37.64, 37.21, 33.46, 33.29, 29.30, 26.83, 26.52, 23.26, 14.80.

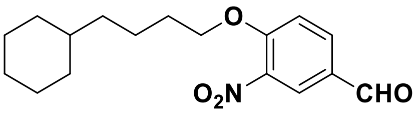

4-(4-Cyclohexylbutoxy)-3-nitrobenzaldehyde (3h) Obtained from the reaction of 4-hydroxy-3-nitrobenzaldehyde and **1**. Yield: 93 %. Yellow oil. ^{1}H NMR (400 MHz, $CDCl_3$) δ 9.91 (s, 1H), 8.32 (s, 1H), 8.05 (d, *J* = 7.3 Hz, 1H), 7.20 (d, *J* = 8.7 Hz, 1H), 4.19 (t, *J* = 6.4 Hz, 2H), 1.88 – 1.79 (m, 2H), 1.71 – 1.55 (m, 6H), 1.27 – 1.09 (m, 7H), 0.91 – 0.79 (m, 2H). ^{13}C NMR (101 MHz, cdcl$_3$) δ 188.93, 156.91, 140.03, 134.67, 128.79, 127.51, 114.60, 70.49, 37.58, 37.07, 33.42, 29.11, 26.78, 26.48, 23.11.

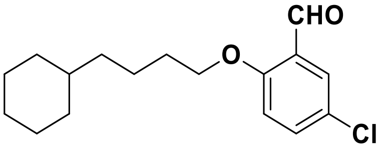 **5-Chloro-2-(4-cyclohexylbutoxy)benzaldehyde (3i)** Obtained from the reaction of 5-chlorosalicylaldehyde and **1**. Yield: 75 %. Yellow oil. ^{1}H NMR (400 MHz, cdcl$_3$) δ 10.41 (s, 1H), 7.74 (d, *J* = 2.8 Hz, 1H), 7.44 (dd, *J* = 8.9, 2.8 Hz, 1H), 6.91 (d, *J* = 8.9 Hz, 1H), 4.04 (t, *J* = 6.4 Hz, 2H), 1.87 – 1.75 (m, 2H), 1.72 – 1.58 (m, 5H), 1.51 – 1.41 (m, 2H), 1.26 – 1.07 (m, 6H), 0.92 – 0.81 (m, 2H). ^{13}C NMR (101 MHz, CDCl$_3$) δ 188.45, 159.94, 135.30, 127.65, 126.05, 125.67, 114.12, 68.99, 37.50, 37.05, 33.31, 29.25, 26.64, 26.33, 23.21.

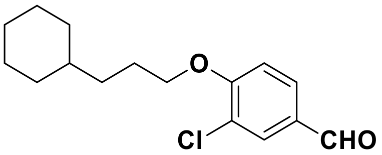 **3-Chloro-4-(3-cyclohexylpropoxy)benzaldehyde (5)** Obtained from the reaction of 3-chloro-4-hydroxybenzaldehyde and **2**. Yield: 93 %. Yellow solid. ^{1}H NMR (400 MHz, CDCl$_3$) δ 9.83 (s, 1H), 7.89 (d, *J* = 2.0 Hz, 1H), 7.73 (dd, *J* = 8.5, 2.0 Hz, 1H), 7.01 (d, *J* = 8.5 Hz, 1H), 4.09 (t, *J* = 6.6 Hz, 2H), 1.94 – 1.83 (m, 2H), 1.83 – 1.56 (m, 5H), 1.45 – 1.08 (m, 6H), 1.00 – 0.84 (m, 2H). ^{13}C NMR (101 MHz, CDCl$_3$) δ 189.63, 159.45, 131.15, 130.39, 129.94, 123.88, 112.41, 69.86, 37.28, 33.48, 33.25, 26.58, 26.48, 26.29, 26.22.

Preparation of the benzylidenenbarbituric acid derivatives 4a~4i and 6

Preparation of 4b.[13,14] The suspension of **3b** (0.30 g, 1.02 mmol, 1.00 equiv.) and barbituric acid (0.13 g, 1.02 mmol, 1.00 equiv.) in absolute ethanol and distilled water (4:1, *v/v*) was heated at reflux for 5 h and the reaction mixture was cooled to room temperature. The precipitate was filtered off, washed with hot water and ethanol, and dried under vacuum to afford the product as yellow powders in high purity (0.37 g, 90 %). The analytical sample was obtained by recrystallization from a mixture of ethanol and DMF. The synthesis of other benzylidenebarbituric acid derivatives (**4a**, **4c~4i**, and **6**) followed the same protocol as described, but with different starting materials.

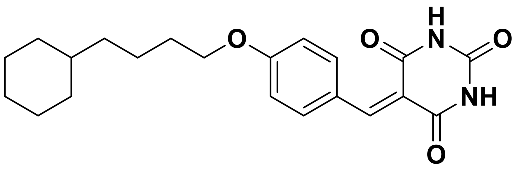 **5-(4-(4-Cyclohexylbutoxy)benzylidene)pyrimidine-2,4,6(*1H,3H,5H*)-trione (4a)** Obtained from the condensation of **3a** with barbituric acid. Yield: 86 %. Yellow solid. ^{1}H NMR (400 MHz, DMSO) δ 11.30 (s, 1H), 11.17 (s, 1H), 8.37 (d, *J* = 8.1 Hz, 2H), 8.25 (s, 1H), 7.05 (d, *J* = 8.0 Hz, 2H), 4.10 (t, *J* = 6.4 Hz, 2H), 1.80 – 1.49 (m, 7H), 1.49 – 1.33 (m, 2H), 1.33 – 0.99 (m, 6H), 0.99 – 0.54 (m, 2H). ^{13}C NMR (101 MHz, DMSO) δ 164.34, 163.40, 162.60, 155.39,

150.61, 137.98, 125.42, 115.81, 114.76, 68.46, 37.42, 36.97, 33.26, 29.20, 26.63, 26.27, 23.12. HRMS (ESI+) *m/z* calcd for $C_{21}H_{27}N_2O_4$ $(MH)^+$: 371.1965, found: 371.1971.

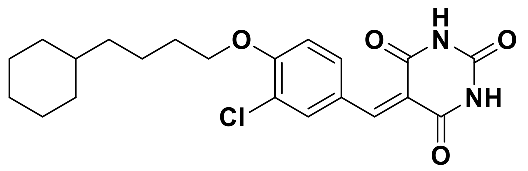

5-(3-Chloro-4-(4-cyclohexylbutoxy)benzylidene)pyrimidine-2,4,6(*1H,3H,5H*)-trione (4b) Obtained from the condensation of **3b** with barbituric acid. Yield: 90 %. Yellow solid. ^{1}H NMR (400 MHz, DMSO) δ 11.34 (s, 1H), 11.23 (s, 1H), 8.66 (d, *J* = 2.1 Hz, 1H), 8.20 (s, 1H), 8.15 (dd, *J* = 9.1, 2.2 Hz, 1H), 7.25 (d, *J* = 8.9 Hz, 1H), 4.18 (t, *J* = 6.4 Hz, 2H), 1.82 – 1.49 (m, 7H), 1.49 – 1.36 (m, 2H), 1.30 – 1.02 (m, 6H), 0.97 – 0.73 (m, 2H). ^{13}C NMR (101 MHz, DMSO) δ 163.58, 162.12, 157.54, 153.26, 150.13, 136.54, 135.20, 125.66, 120.96, 116.92, 112.94, 69.09, 36.94, 36.41, 32.80, 28.57, 26.20, 25.83, 22.59. HRMS (ESI+) *m/z* calcd for $C_{21}H_{26}BrN_2O_4$ $(MH)^+$: 405.1576, found: 405.1574.

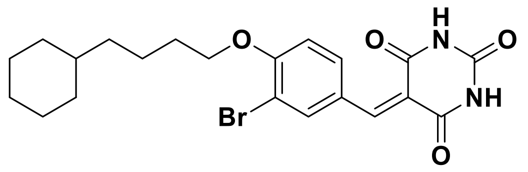

5-(3-Bromo-4-(4-cyclohexylbutoxy)benzylidene)pyrimidine-2,4,6(*1H,3H,5H*)-trione (4c) Obtained from the condensation of **3c** with barbituric acid. Yield: 92 %. Yellow solid. ^{1}H NMR (400 MHz, DMSO) δ 11.34 (s, 1H), 11.22 (s, 1H), 8.81 (d, *J* = 2.1 Hz, 1H), 8.19 (dd, *J* = 8.2, 2.7 Hz, 2H), 7.21 (d, *J* = 8.9 Hz, 1H), 4.17 (t, *J* = 6.3 Hz, 2H), 1.78 – 1.58 (m, 7H), 1.50 – 1.41 (m, 2H), 1.26 – 1.05 (m, 6H), 0.90 – 0.79 (m, 2H). ^{13}C NMR (101 MHz, DMSO) δ 163.57, 162.12, 158.38, 153.15, 150.13, 138.34, 137.09, 126.19, 116.87, 112.73, 110.60, 69.14, 36.95, 36.40, 32.80, 28.57, 26.20, 25.83, 22.61. HRMS (ESI+) *m/z* calcd for $C_{21}H_{26}BrN_2O_4$ $(MH)^+$: 449.1070, found: 449.1059.

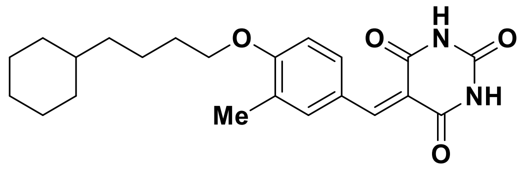

5-(4-(4-Cyclohexylbutoxy)-3-methylbenzylidene)pyrimidine-2,4,6(*1H,3H,5H*)-trione (4d) Obtained from the condensation of **3d** with barbituric acid. Yield: 82 %. Orange solid. ^{1}H NMR (400 MHz, DMSO) δ 11.27 (s, 1H), 11.14 (s, 1H), 8.30 (dd, *J* = 8.8, 2.1 Hz, 1H), 8.21 (s, 2H), 7.05 (d, *J* = 8.8 Hz, 1H), 4.10 (t, *J* = 6.3 Hz, 2H), 2.17 (s, 3H), 1.77 – 1.57 (m, 7H), 1.49 – 1.39 (m, 2H), 1.25 – 1.04 (m, 6H), 0.90 – 0.79 (m, 2H). ^{13}C NMR (101 MHz, DMSO) δ 163.96, 162.15, 161.34, 155.30, 150.19, 137.51, 135.98, 125.63, 124.56, 114.97, 110.88, 68.00, 36.95, 36.50, 32.81, 28.77, 26.20, 25.84, 22.72, 15.88. HRMS (ESI+) *m/z* calcd for $C_{22}H_{29}N2O_4$ $(MH)^+$: 385.2122, found: 385.2125.

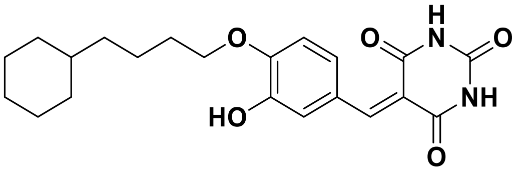

5-(4-(4-Cyclohexylbutoxy)-3-hydroxybenzylidene)pyrimidine-2,4,6(*1H,3H,5H*)-trione (4e) Obtained from the condensation of **3e** with barbituric acid. Yield: 86 %. Yellow solid. ^{1}H NMR (400 MHz, DMSO) δ 11.26 (s, 1H), 11.14 (s, 1H), 9.32 (s, 1H), 8.14 (d, *J* = 4.9 Hz, 2H), 7.71 (d, *J* = 8.5 Hz, 1H), 7.04 (d, *J* = 8.0 Hz, 1H), 4.08 (t, *J* = 5.6 Hz, 2H), 1.78 – 1.57 (m, 7H), 1.49 – 1.38 (m, 2H), 1.28 – 1.05 (m, 6H), 0.93 – 0.79 (m, 2H). ^{13}C NMR (101 MHz, DMSO) δ 164.00, 162.11, 155.55, 152.39, 150.19, 145.86, 130.37, 125.18, 120.49, 114.86, 112.05, 68.32, 37.04, 36.59, 32.84, 28.84, 26.21, 25.85, 22.70. HRMS (ESI+) *m/z* calcd for $C_{21}H_{27}N_2O_5$ $(MH)^+$: 387.1914, found: 387.1920.

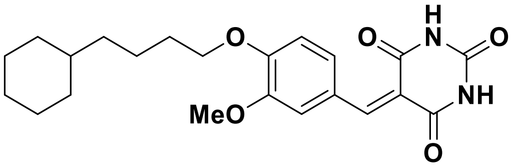

5-(4-(4-Cyclohexylbutoxy)-3-methoxybenzylidene)pyrimidine-2,4,6(*1H,3H,5H*)-trione (4f) Obtained from the condensation of **3f** with barbituric acid. Yield: 95 %. Yellow solid. ^{1}H NMR (400 MHz, DMSO) δ 11.30 (s, 1H), 11.17 (s, 1H), 8.41 (s, 1H), 8.24 (s, 1H), 7.87 (d, *J* = 8.5 Hz, 1H), 7.08 (d, *J* = 8.5 Hz, 1H), 4.07 (t, *J* = 5.9 Hz, 2H), 3.81 (s, 3H), 1.77 – 1.54 (m, 7H), 1.46 – 1.03 (m, 8H), 0.92 – 0.77 (m, 2H). ^{13}C NMR (101 MHz, DMSO) δ 163.99, 162.36, 155.50, 153.19, 150.17, 147.87, 131.80, 125.11, 117.04, 115.06, 111.79, 68.43, 55.45, 36.99, 36.53, 32.83, 28.77, 26.22, 25.85, 22.73. HRMS (ESI+) *m/z* calcd for $C_{22}H_{29}N_2O_5$ $(MH)^+$: 401.2071, found: 401.2074.

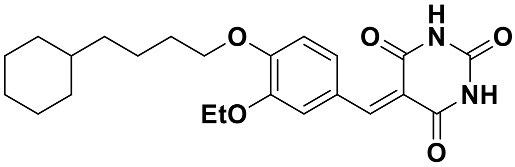

5-(4-(4-Cyclohexylbutoxy)-3-ethoxybenzylidene)pyrimidine-2,4,6(*1H,3H,5H*)-trione (4g) Obtained from the condensation of **3g** with barbituric acid. Yield: 92 %. Yellow solid. ^{1}H NMR (400 MHz, DMSO) δ 11.28 (d, *J* = 1.3 Hz, 1H), 11.16 (d, *J* = 1.4 Hz, 1H), 8.42 (d, *J* = 2.1 Hz, 1H), 8.23 (s, 1H), 7.82 (dd, *J* = 8.7, 2.0 Hz, 1H), 7.08 (d, *J* = 8.7 Hz, 1H), 4.12 – 4.02 (m, 4H), 1.75 – 1.56 (m, 7H), 1.46 – 1.32 (m, 5H), 1.25 – 1.03 (m, 6H), 0.96 – 0.72 (m, 2H). ^{13}C NMR (101 MHz, DMSO) δ 163.96, 162.35, 155.53, 153.46, 150.14, 147.07, 131.88, 125.12, 118.29, 114.99, 112.05, 68.39, 63.82, 36.95, 36.46, 32.79, 28.70, 26.21, 25.84, 22.64, 14.58. HRMS (ESI+) *m/z* calcd for $C_{23}H_{31}N_2O_5$ $(MH)^+$: 415.2227, found: 415.2231.

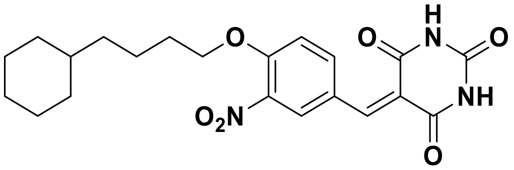

5-(4-(4-Cyclohexylbutoxy)-3-nitrobenzylidene)pyrimidine-2,4,6(*1H,3H,5H*)-trione (4h) Obtained from the condensation of **3h** with barbituric acid. Yield: 94 %. Yellow solid. ^{1}H NMR (400 MHz, dmso) δ 11.40 (s, 1H), 11.29 (s, 1H), 8.99 (d, *J* = 2.1 Hz, 1H), 8.37 (dd, *J* = 9.2, 2.2 Hz, 1H), 8.25 (s, 1H), 7.45 (d, *J* = 9.0 Hz, 1H), 4.26 (t, *J* = 6.3 Hz, 2H), 1.75 – 1.56 (m, 7H), 1.47 – 1.37 (m, 2H), 1.25 – 1.06 (m, 6H), 0.84 (dd, *J* = 20.8, 10.4 Hz, 2H). ^{13}C NMR (101 MHz, DMSO) δ 163.73, 162.48, 154.62, 152.44, 150.57, 141.09, 139.09, 130.47, 124.99, 118.90, 114.90, 70.22, 37.36, 36.77, 33.21, 28.87, 26.64, 26.26, 22.89. HRMS (ESI+) *m/z* calcd for $C_{21}H_{29}N_4O_6$ $(M+NH_4)^+$: 433.2082, found: 433.2084.

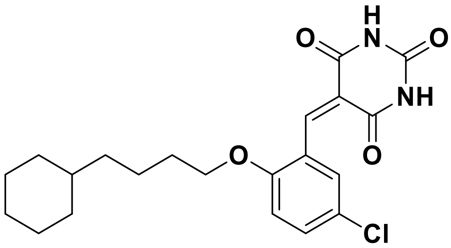

5-(5-Chloro-2-(4-cyclohexylbutoxy)benzylidene)pyrimidine-2,4,6(*1H,3H,5H*)-trione (4i) Obtained from the condensation of **3i** with barbituric acid. Yield: 73 %. Yellow solid. ^{1}H NMR (400 MHz, DMSO) δ 11.39 (s, 1H), 11.21 (s, 1H), 8.38 (s, 1H), 8.00 (d, *J* = 2.6 Hz, 1H), 7.50 (dd, *J* = 8.9, 2.7 Hz, 1H), 7.11 (d, *J* = 9.0 Hz, 1H), 4.06 (t, *J* = 6.3 Hz, 2H), 1.79 – 1.47 (m, 7H), 1.47 – 1.32 (m, 2H), 1.32 – 0.97 (m, 6H), 0.96 – 0.48 (m, 2H). ^{13}C NMR (101 MHz, DMSO) δ 162.98, 161.41, 156.96, 150.17, 147.86, 132.78, 131.31, 123.31, 123.14, 119.98, 113.72, 68.75, 36.95, 36.48, 32.77, 28.67, 26.20, 25.83, 22.67. HRMS (ESI+) *m/z* calcd for $C_{21}H_{26}ClN_2O_4$ $(MH)^+$: 405.1576, found: 405.1578.

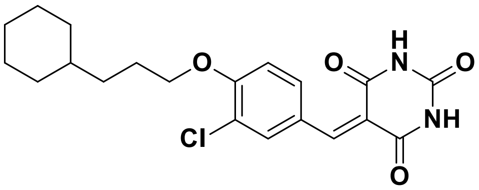

5-(3-Chloro-4-(3-cyclohexylpropoxy)benzylidene)pyrimidine-2,4,6(*1H,3H,5H*)-trione (6) Obtained from the condensation of **5** with barbituric acid. Yield: 94 %. Yellow solid. ^{1}H NMR (400 MHz, DMSO) δ 11.34 (s, 1H), 11.23 (s, 1H), 8.66 (d, *J* = 2.1 Hz, 1H), 8.20 (s, 1H), 8.15 (dd, *J* = 9.1, 2.2 Hz, 1H), 7.25 (d, *J* = 8.9 Hz, 1H), 4.17 (t, *J* = 6.4 Hz, 2H), 1.87 – 1.50 (m, 7H), 1.40 – 1.05 (m, 6H), 0.98 – 0.75 (m, 2H). ^{13}C NMR (101 MHz, DMSO) δ 163.58, 162.12, 157.53, 153.24, 150.13, 136.53, 135.20, 125.66, 120.95, 116.93, 112.94, 109.55, 69.44, 36.62, 33.07, 32.80, 26.15, 25.78, 25.72. HRMS (ESI+) *m/z* calcd for $C_{20}H_{24}ClN_2O_4$ $(MH)^+$: 391.1419, found: 391.1417.

Purity data of final products (4a~4i and 6) as determined by HPLC

Purity was determined by HPLC (Waters 1525 Binary HPLC pump, Waters 2487 Dual λ Absorbance Detector, and Waters 717plus Autosampler, Milford, MA) at λ of 370 nM using acetonitrile and 0.1% TFA (70:30) as mobile phase. All the final products described here were verified to have a purity of 95% or higher.

Compound	Purity (%)	t_R (min)
**4a**	99.8	11.824
**4b**	98.0	14.436
**4c**	97.9	15.124
**4d**	98.6	15.845
**4e**	97.6	7.437
**4f**	99.9	10.023
**4g**	99.9	12.199
**4h**	99.1	10.313
**4i**	99.7	11.805
**6**	99.5	11.383

3. *In vitro* experimental tests

Preparation of mPGES-1 enzymes. Cloning of mPGES-1 and the protein preparation were described in our previous reports.[15] Briefly, FreeStyle Max Expression system was used to express wild-type human and mouse mPGES-1 enzymes separately. FreeStyle 293-F cells were cultured following manufacturer's manual in FreeStyle 293 expression medium on orbit rotate shaker in 8% CO_2 incubator at 37°C. Cells were transfected with 1.5 μg/mL of mPGES-1/pcDNA3 construct using FreeStyle Max reagent at a cell density of 1×10^6 for 2 days. Transfected cells were collected, washed, and sonicated in TSES buffer (15 mM Tris-HCl, pH 8.0 plus 0.25 M sucrose, 0.1 mM EDTA and 1 mM DTT) on ice. The broken cells were first centrifuged at 12,500 × *g* for 10 min. The supernatant was further centrifuged at 105,000 × *g* for 1 hr at 4°C. The pellet was washed and homogenized in PBS buffer. The crude microsomal mPGES-1 preparations were aliquoted and stored at -80 °C before use.

Activity assays using a recombinant mPGES-1. The enzyme activity assays were performed according to our previous reports.[15,16] Briefly, the mPGES-1-catalyzed reaction was carried out in 1.5 mL microfuge tubes with reaction mixture of 0.2 Na_2HPO_4/NaH_2PO_4, pH 7.2 (10 µL); 2.5 mM GSH (2.5 µL); diluted microsomal human or mouse mPGES-1 enzyme (80 µg/mL, 1 µL); inhibitor in DMSO solution (1 µL); 0.31 mM PGH_2 in DMF (5 µL) and distilled deionized water in a final volume of 100 µL. An inhibitor was incubated with enzyme for 15 min at ambient temperature followed by the addition of cold PGH_2 (stored in dry ice). The enzymatic reaction was initiated immediately upon adding PGH_2. After 1 min of reaction, stop solution (40 mg/mL $SnCl_2$ in absolute ethanol, 10 µL) was added to cease the reaction by converting excess PGH_2 to $PGF_{2\alpha}$. The produced PGE_2 from the enzymatic reaction was quantified by the PGE_2 enzyme immunoassay as described earlier.[17]

Activity assays of COX-1/2. The inhibition of COX isoenzymes was determined by using COX (ovine/human) inhibitor screening assay kit purchased from Cayman Chemical Company (Ann Arbor, MI). We used a mixture of equal amount COX-1 and COX-2 instead of single enzyme and followed the recommended protocol.

4. *In vivo* experiments

Animals. Wild-type CD-1 mice (28-35 g) were ordered from Harlan (Indianapolis, IN), and housed for a week prior to the experimental studies. All animals were allowed ad libitum access to food and water and maintained on a 12 h light/12 h dark cycle, with the lights on at 8:00 am at a room temperature of 21–22 ^{o}C. Experiments were performed in a same colony room in accordance with the Guide for the Care and Use of Laboratory Animals as adopted and promulgated by the National Institutes of Health. The animal protocol was approved by the IACUC (Institutional Animal Care and Use Committee) at the University of Kentucky.

Wild-type mice based air-pouch model of inflammation. Air pouches (cavities) were produced by duplicate injections of 3 mL of sterile air under the skin on the backs of mice. Following the formation of the air-pouch, six days after the initial air injection, 1 mL of 1% (w/v) solution of carrageenan dissolved in saline was injected directly into the pouch to produce an inflammatory response. Then, the mice were treated SC (subcutaneous) or PO (oral gavage) with a single dose of **4b**, celecoxib, or vehicle. 24 hours later, the mice were sacrificed by carbon dioxide (compressed gas) inhalation until visible respiration has ceased, followed by thoracotomy, and the air-pouch fluid and other tissues (including kidney) were collected. The collected samples were analyzed for their PGE_2 concentrations by using the same ELISA method described above.

Preparation of pictures during acute toxicity testing. Mice were divided into three groups and treated with oral administration of a single dose of vehicle (Wesson vegetable oil, Memphis, TN), **4b** (250 mg/mL in Wesson vegetable oil at the dose of 1 g/kg), and celecoxib (12.5 mg/mL in Wesson vegetable

oil at the dose of 50 mg/kg). After 24 hours, the stomach tissues were dissected, washed with distilled water, and stored at -80 ^{o}C before analysis. The stomach samples were thawed out, fixed in formalin, and rinsed with saline, and the pictures were taken under the microscope Olympus SZX9 (Olympus America, Center Valley, PA).

5. ^{1}H and ^{13}C NMR spectra of products 4a~4i and 6

4a

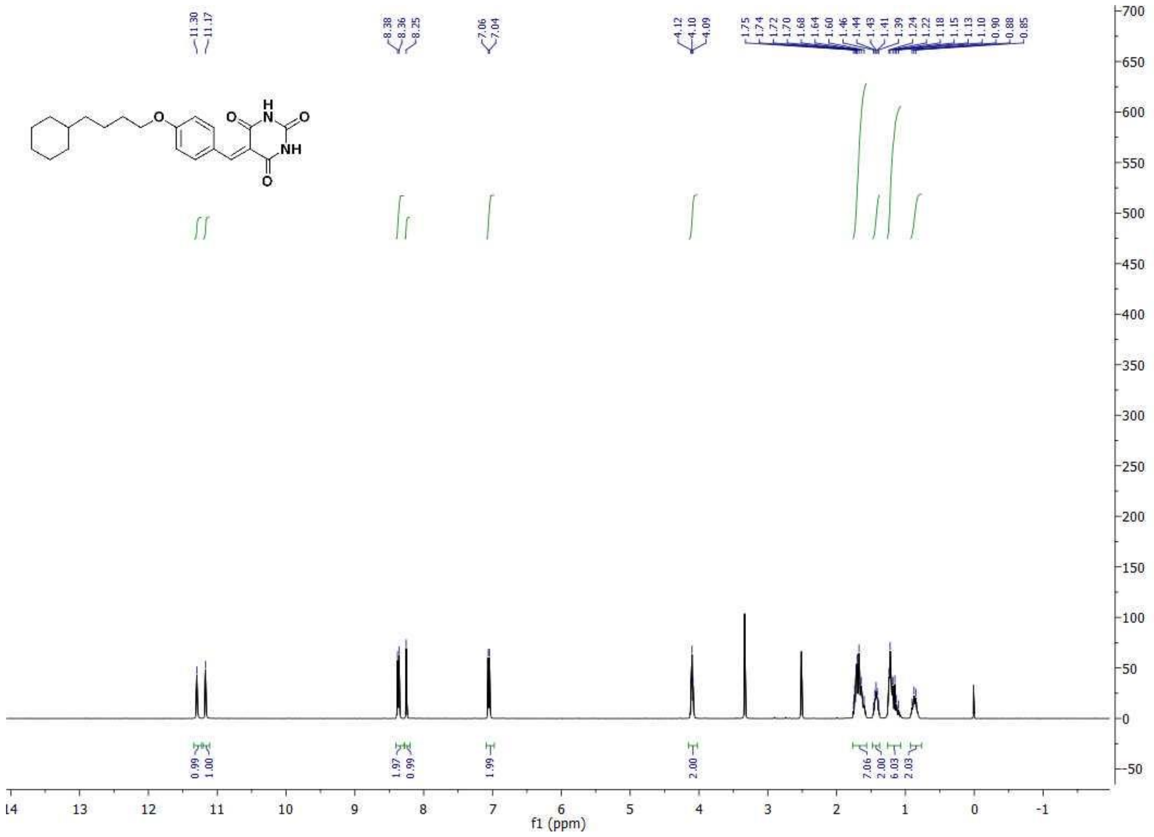

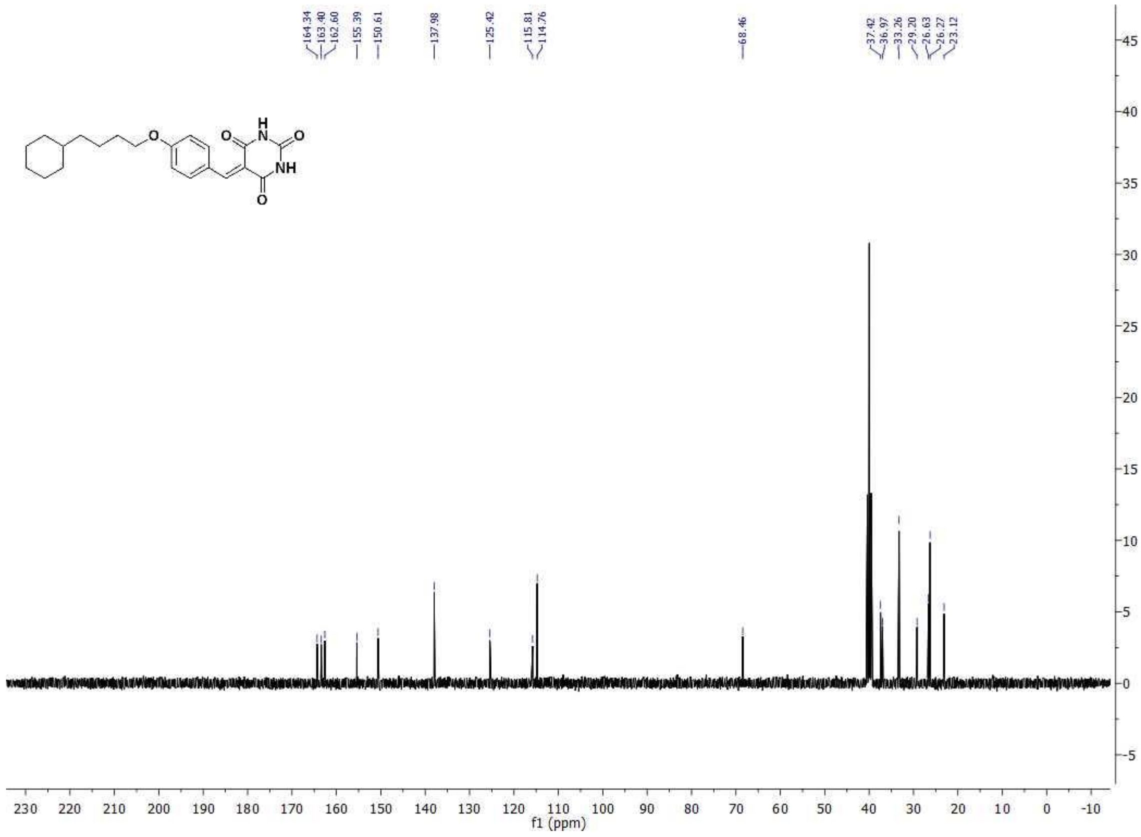

164.34
163.40
162.60
155.39
150.61
137.98
125.42
115.81
114.76
68.46
37.42
36.97
33.26
29.20
26.63
26.27
23.12
f1 (ppm)

4b

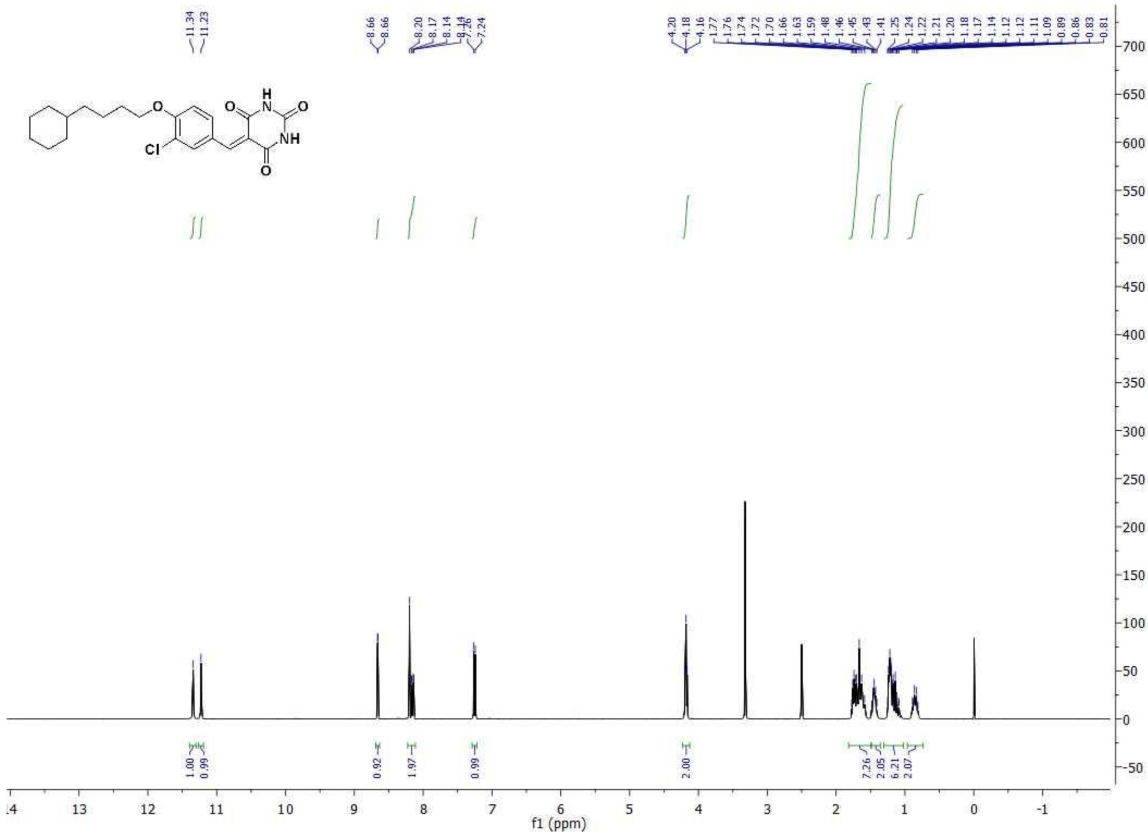

11.34
11.23
8.66
8.66
8.20
8.17
8.14
7.26
7.24
4.20
4.18
4.16
Cl
f1 (ppm)

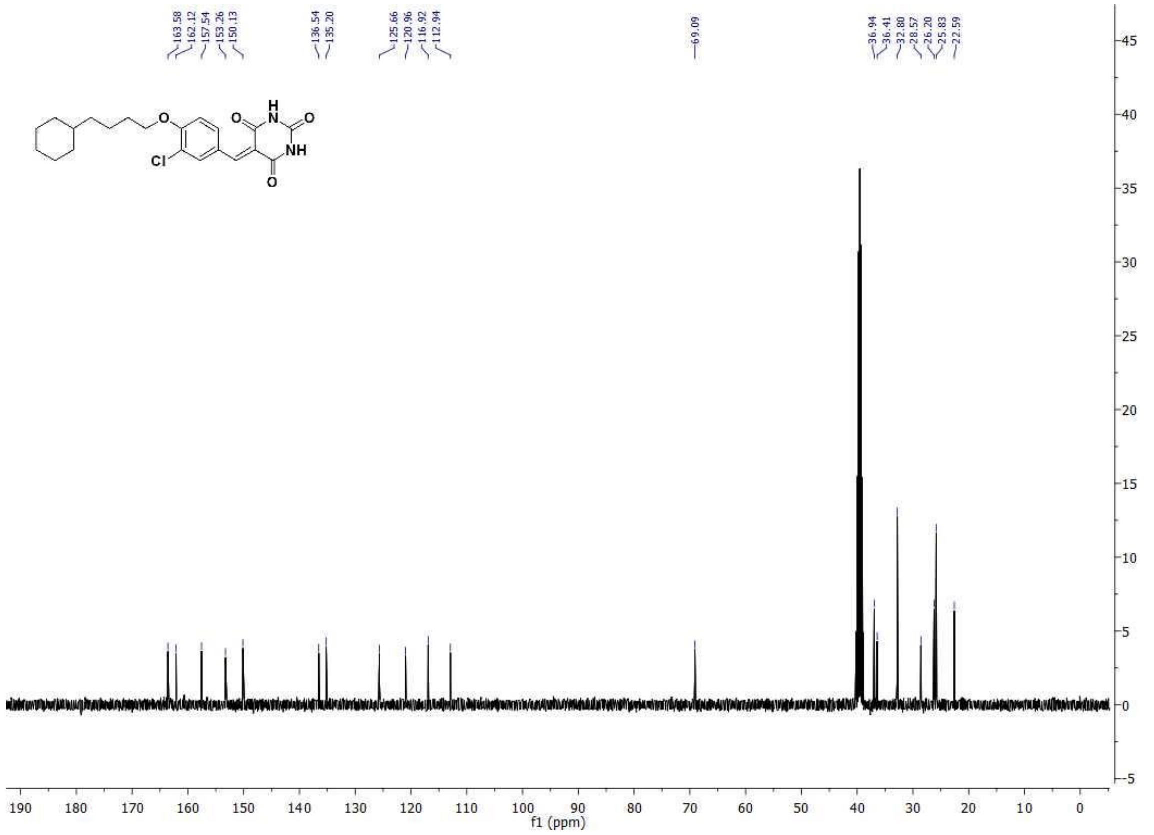

4c

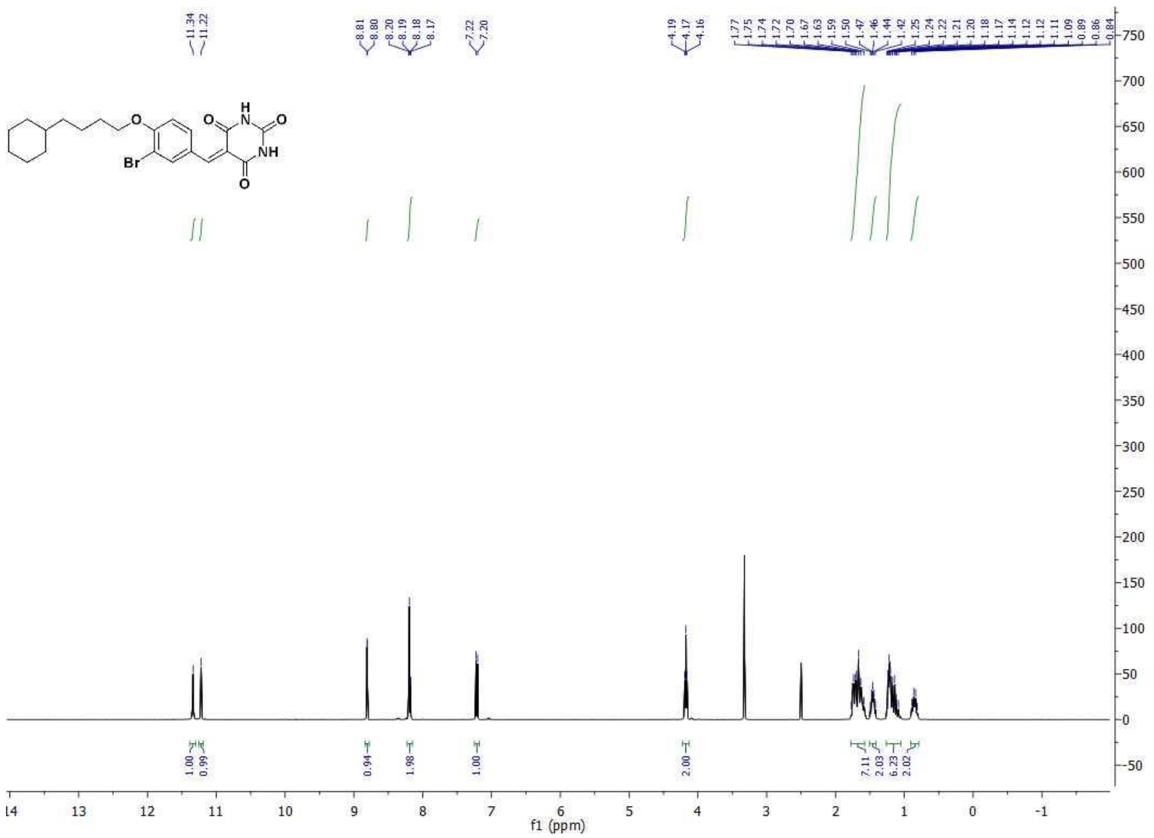

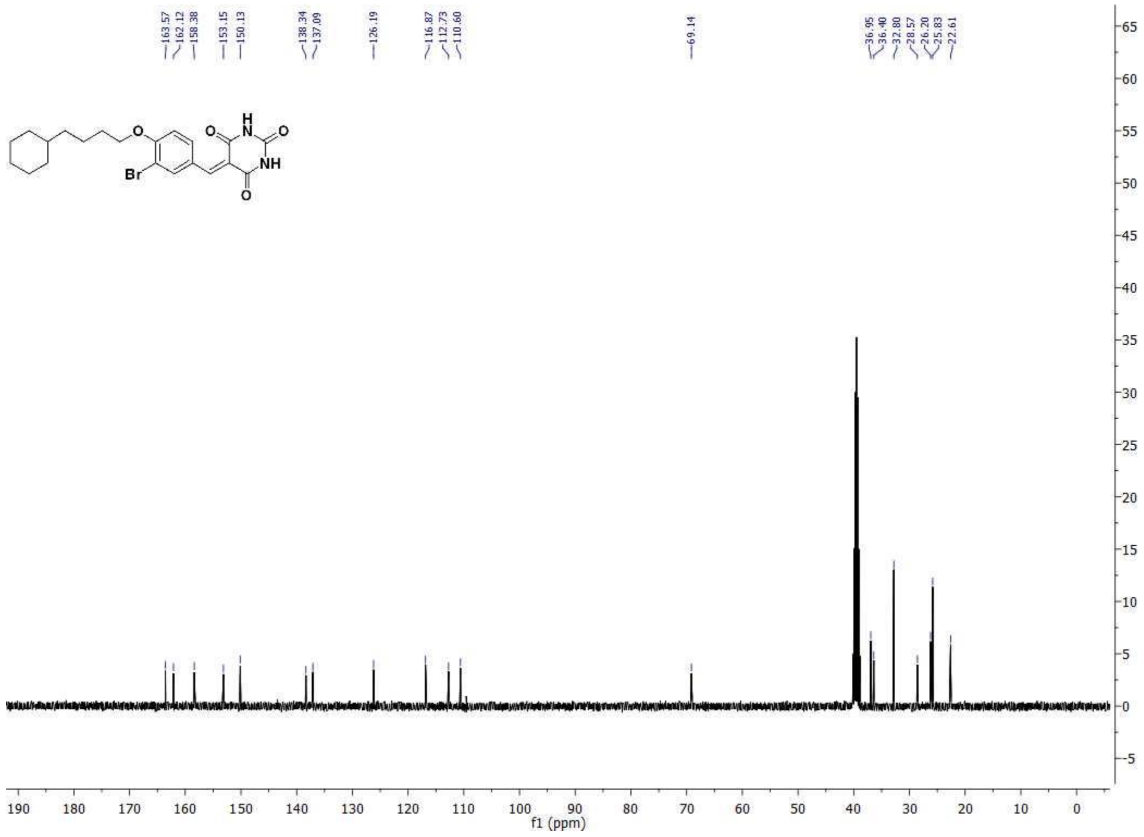

4d

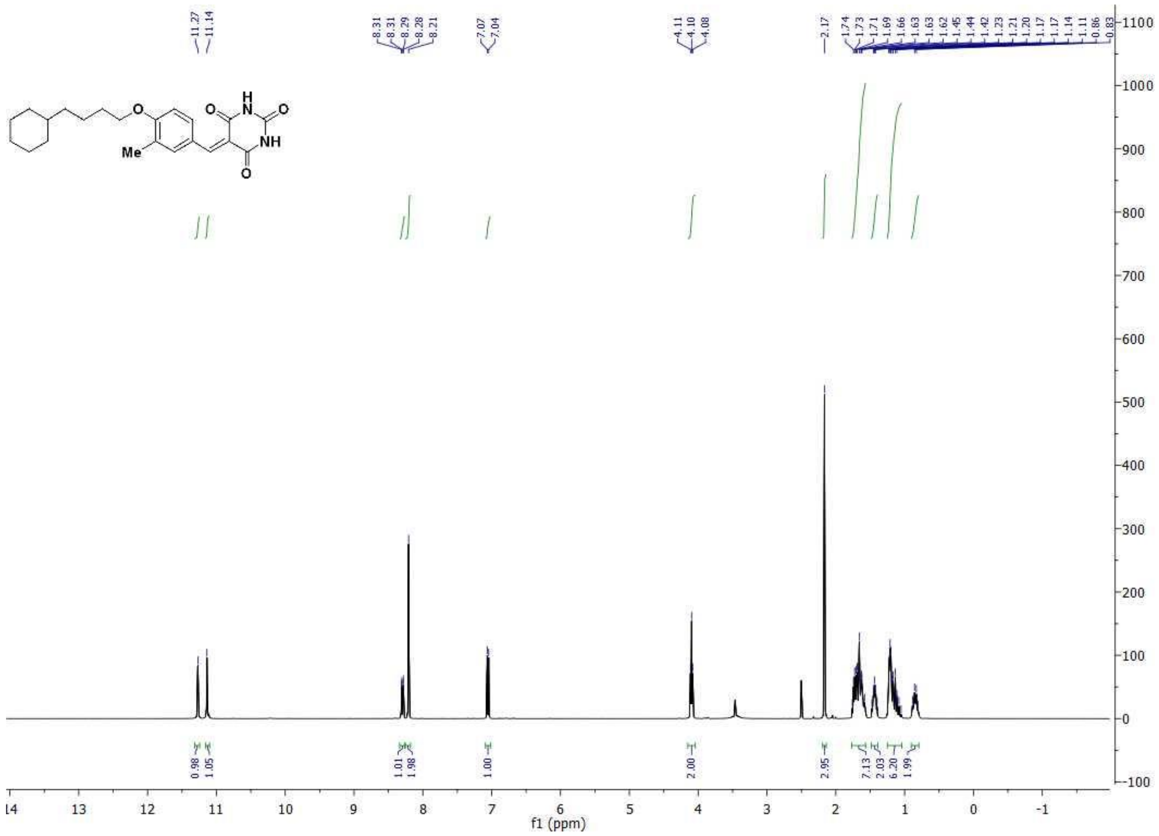

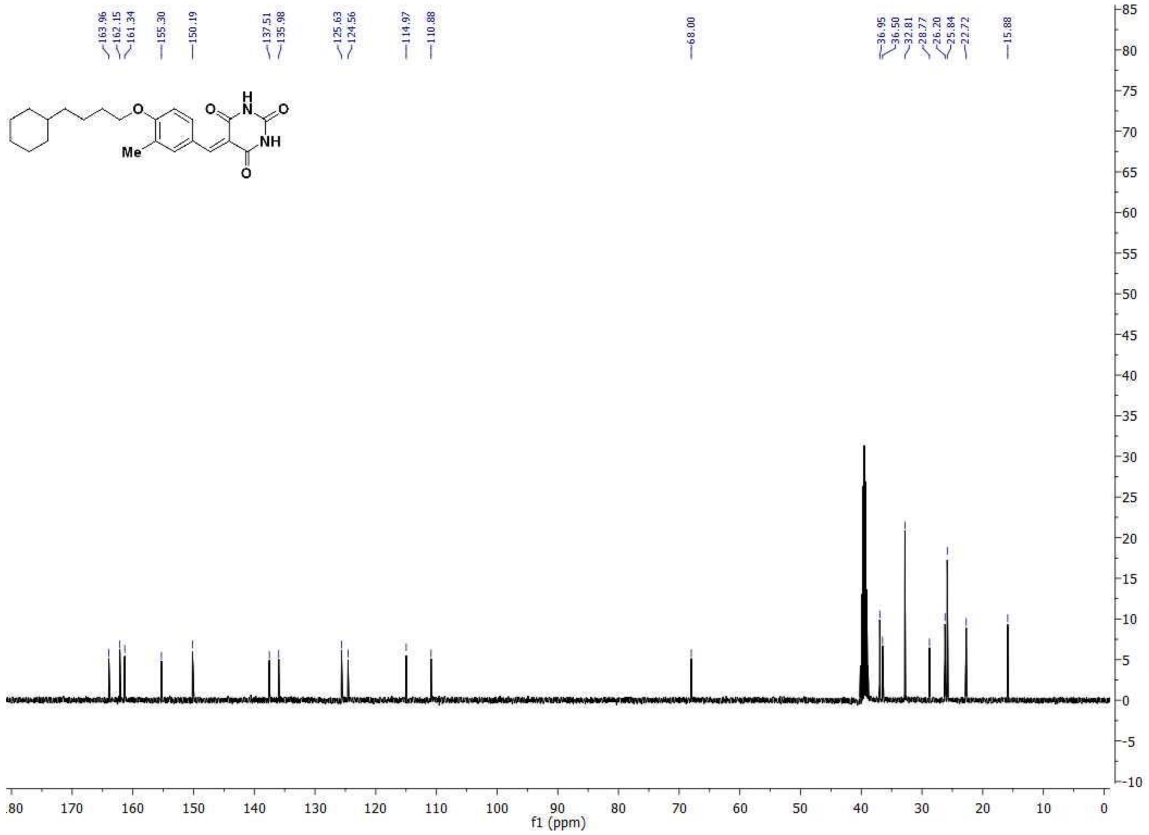

163.96
162.15
161.34
155.30
150.19
137.51
135.98
125.63
124.56
114.97
110.88
68.00
36.95
36.50
32.81
28.77
26.20
25.84
22.72
15.88
Me
f1 (ppm)

4e

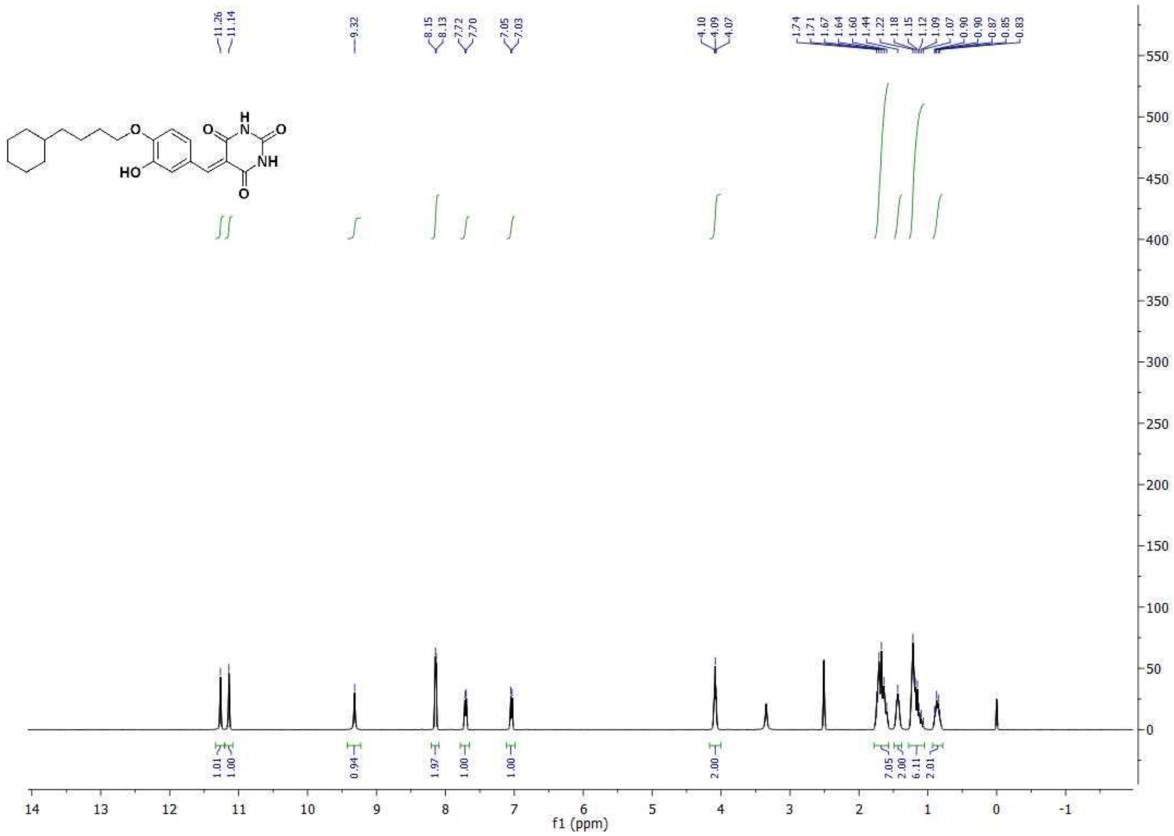

11.26
11.14
9.32
8.15
8.13
7.72
7.70
7.05
7.03
4.10
4.09
4.07
1.74
1.71
1.67
1.64
1.60
1.44
1.22
1.18
1.15
1.12
1.09
1.07
0.90
0.90
0.87
0.85
0.83
HO
1.01
1.00
0.94
1.97
1.00
1.00
2.00
7.05
2.00
6.11
2.01
f1 (ppm)

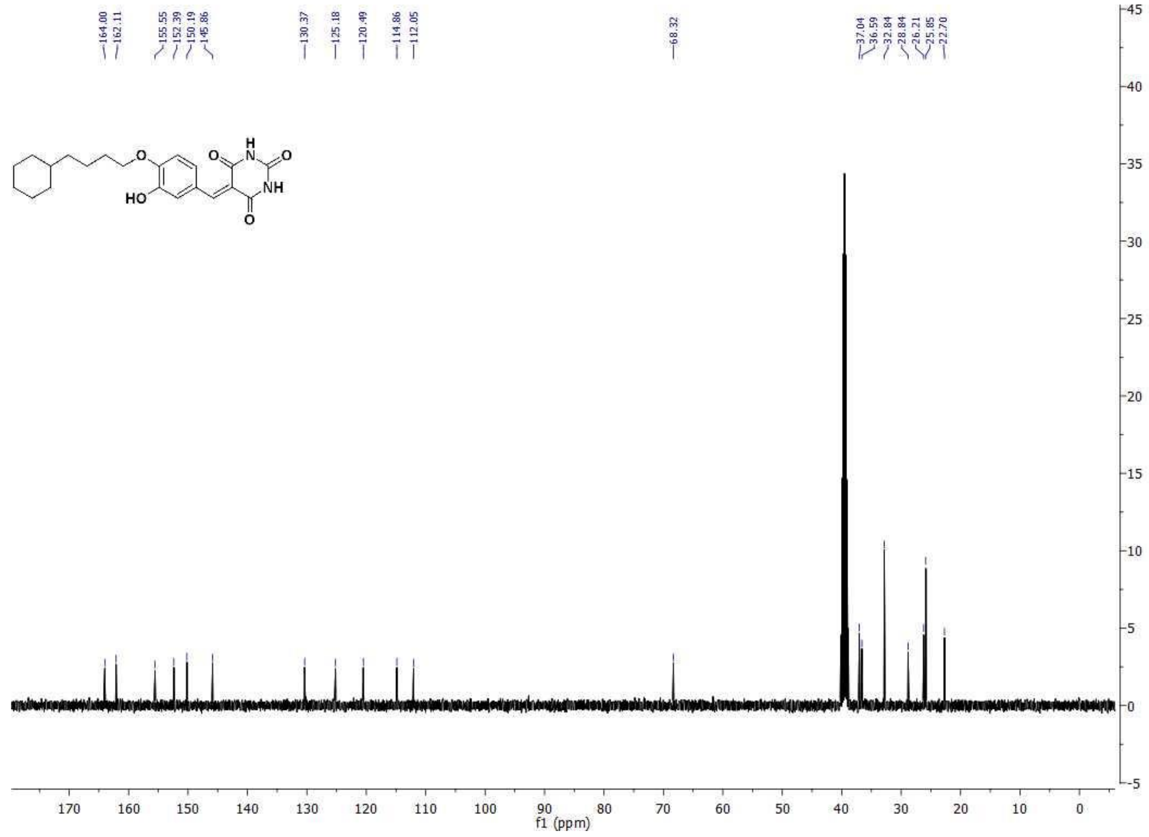

4f

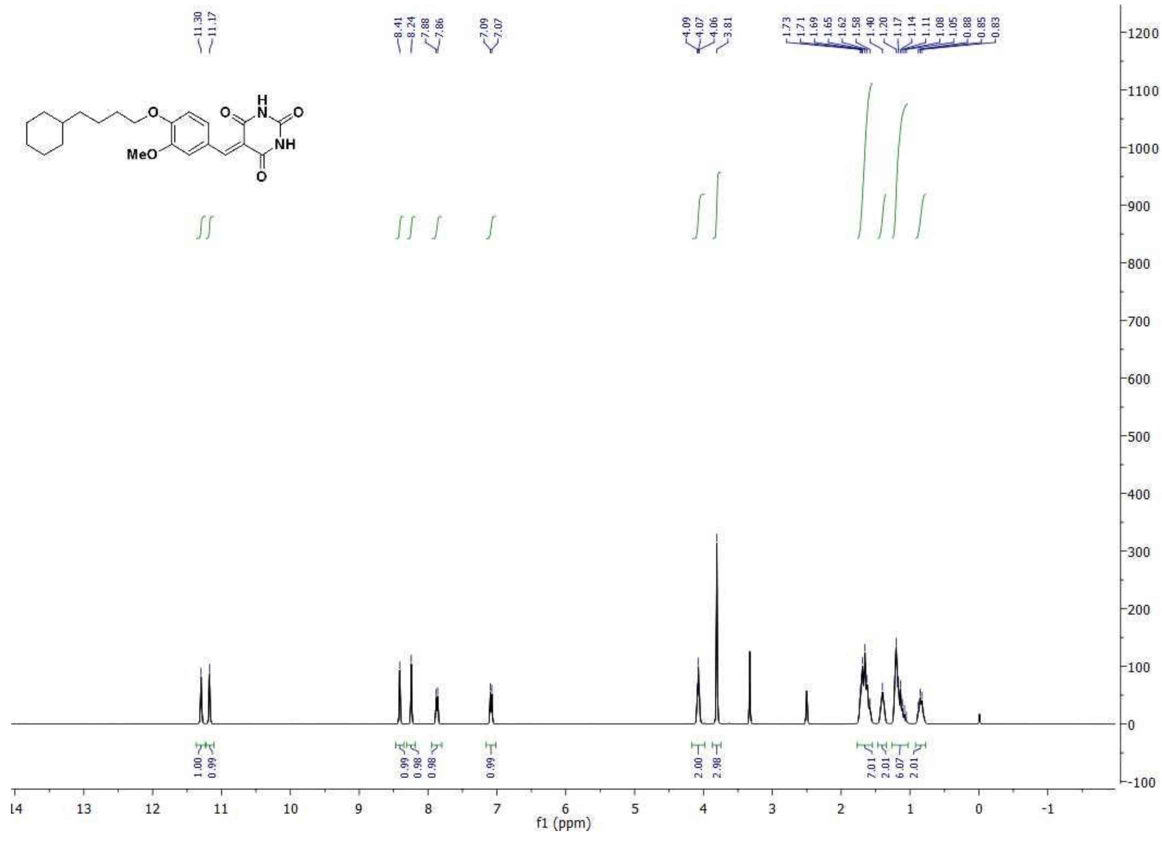

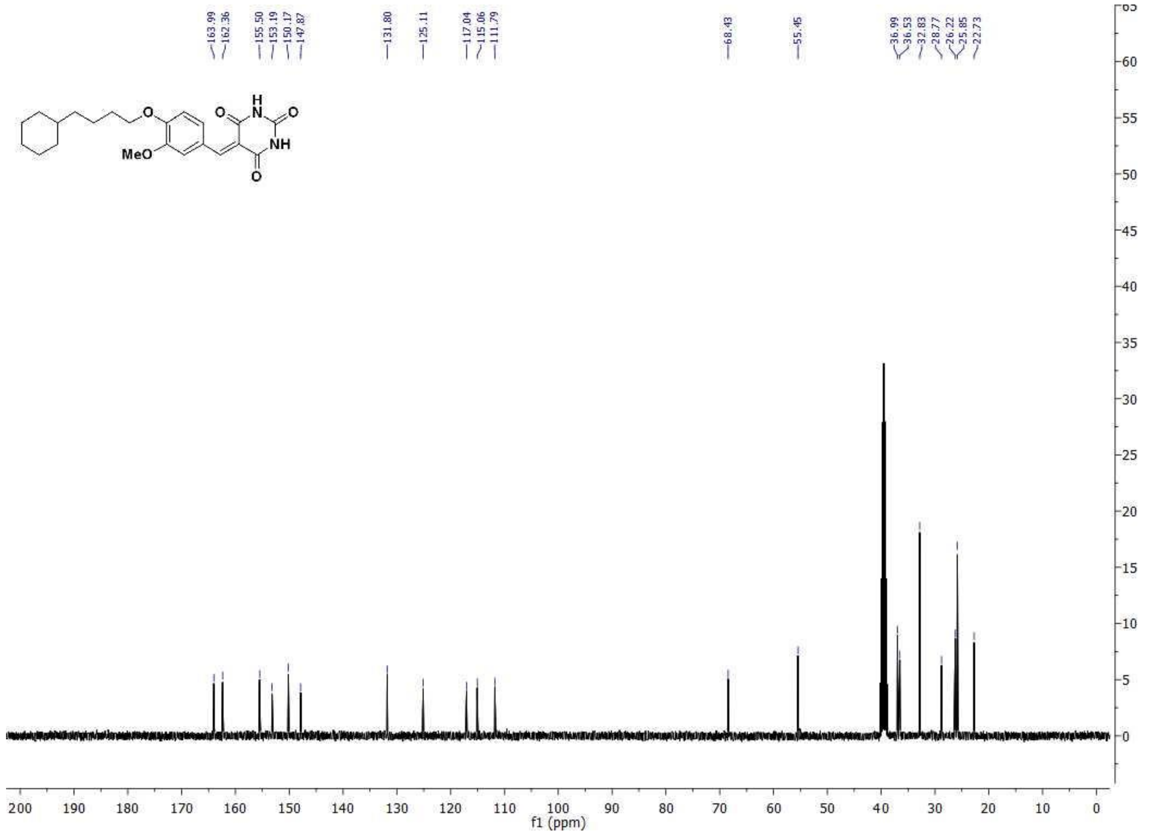

163.99
162.36
155.50
153.19
150.17
147.87
131.80
125.11
117.04
115.06
111.79
68.43
55.45
36.99
36.53
32.83
28.77
26.22
25.85
22.73
MeO
NH
f1 (ppm)

4g

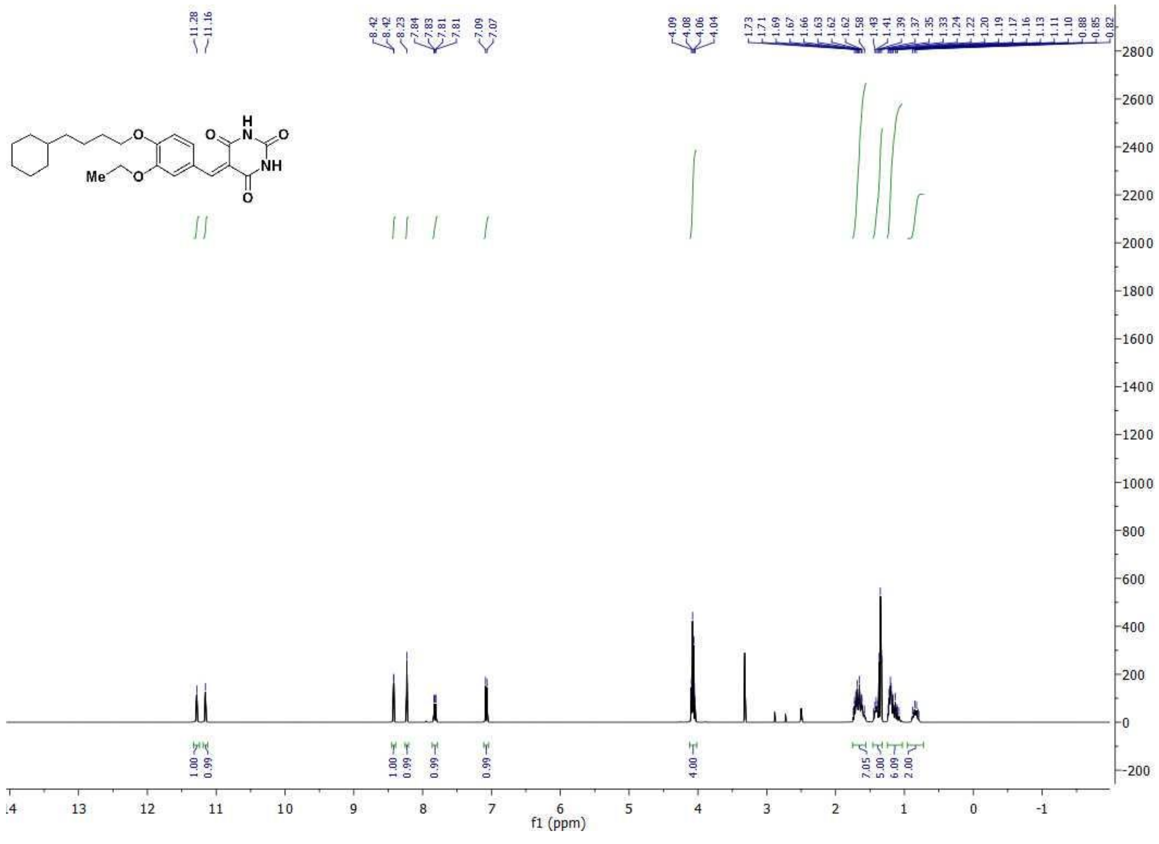

11.28
11.16
8.42
8.42
8.23
7.84
7.83
7.81
7.81
7.09
7.07
4.09
4.08
4.06
4.04
Me
NH
f1 (ppm)

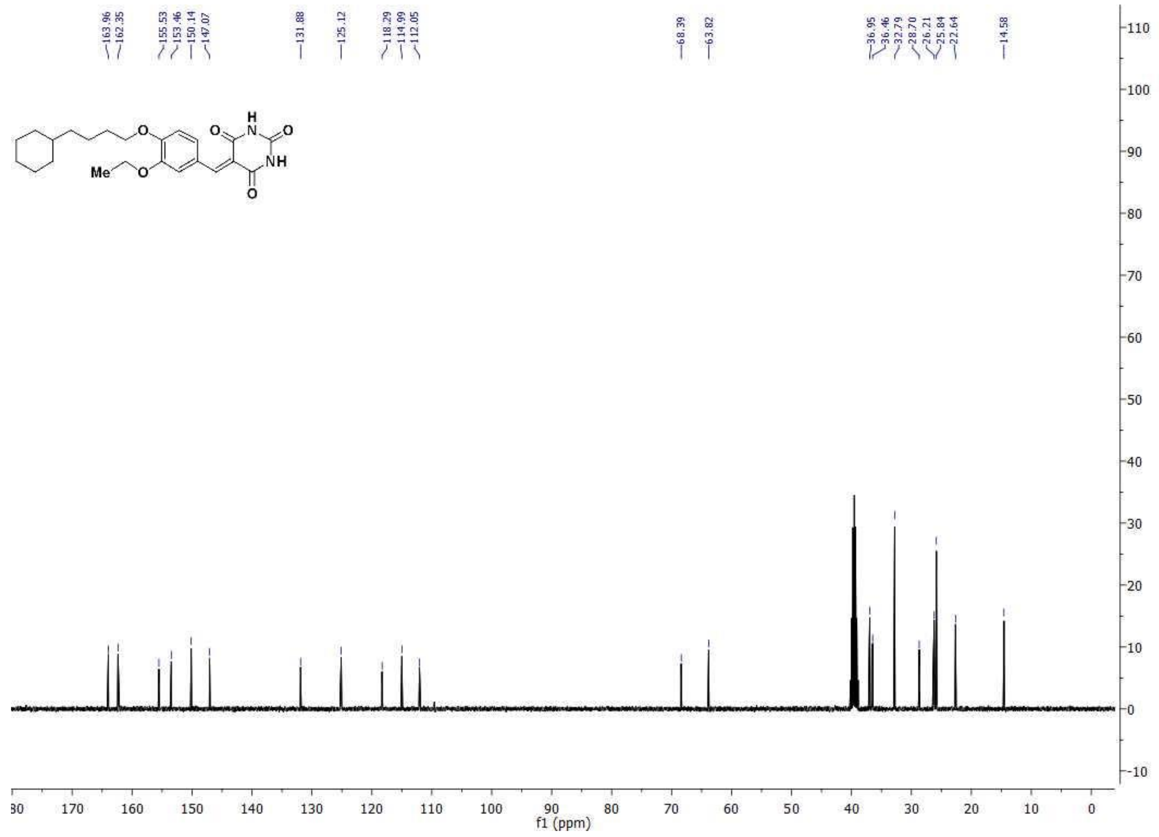

4h

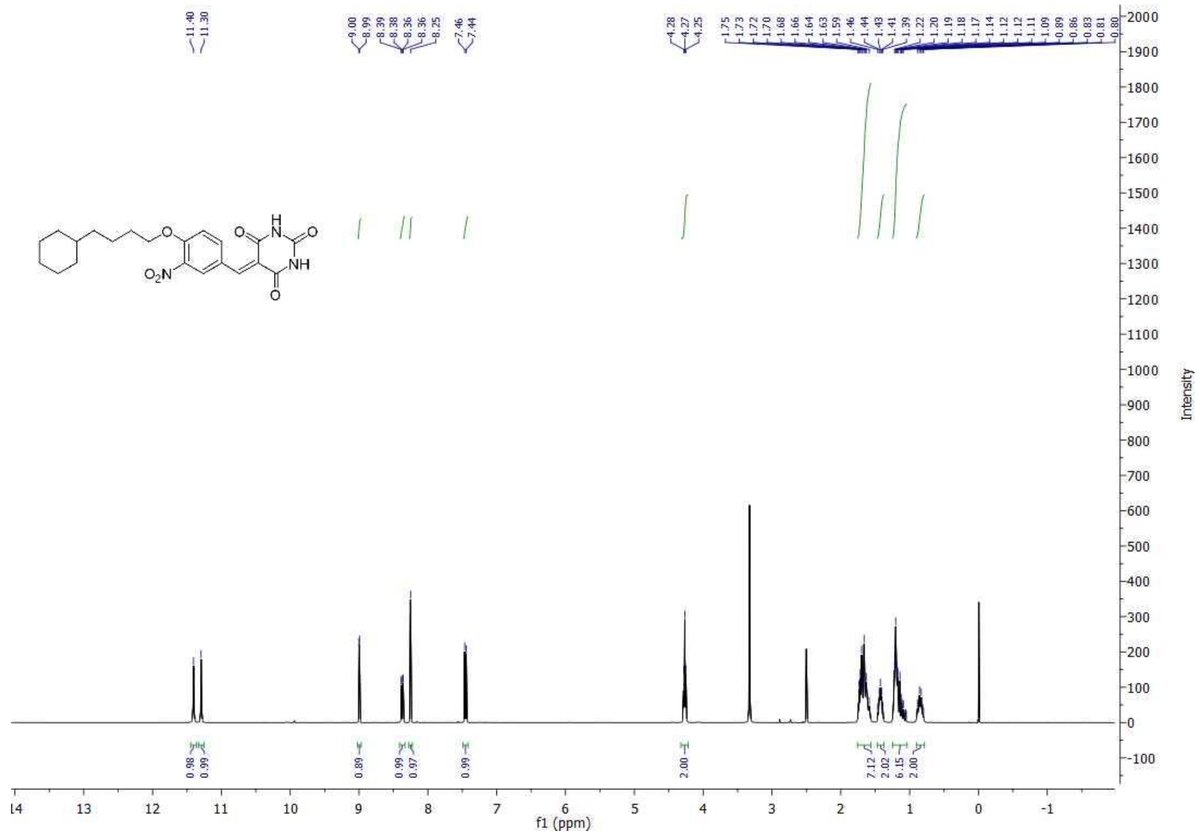

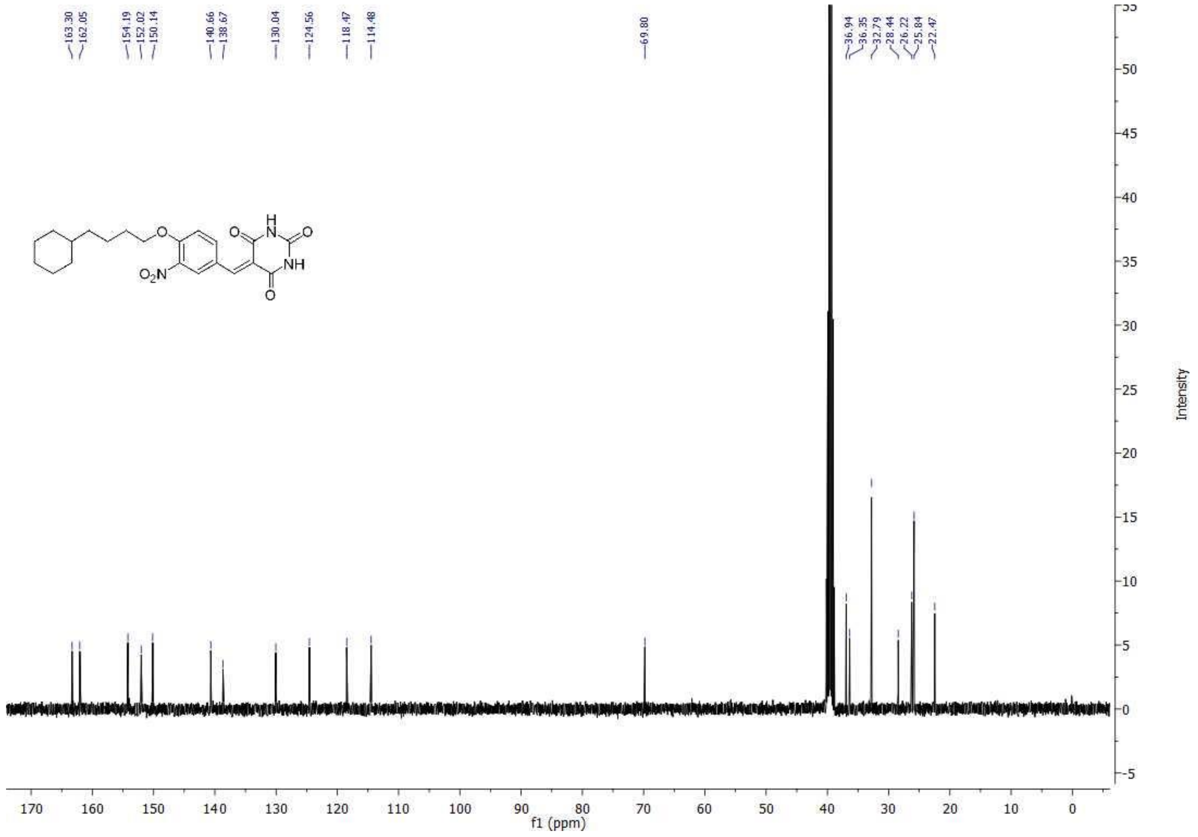

4i

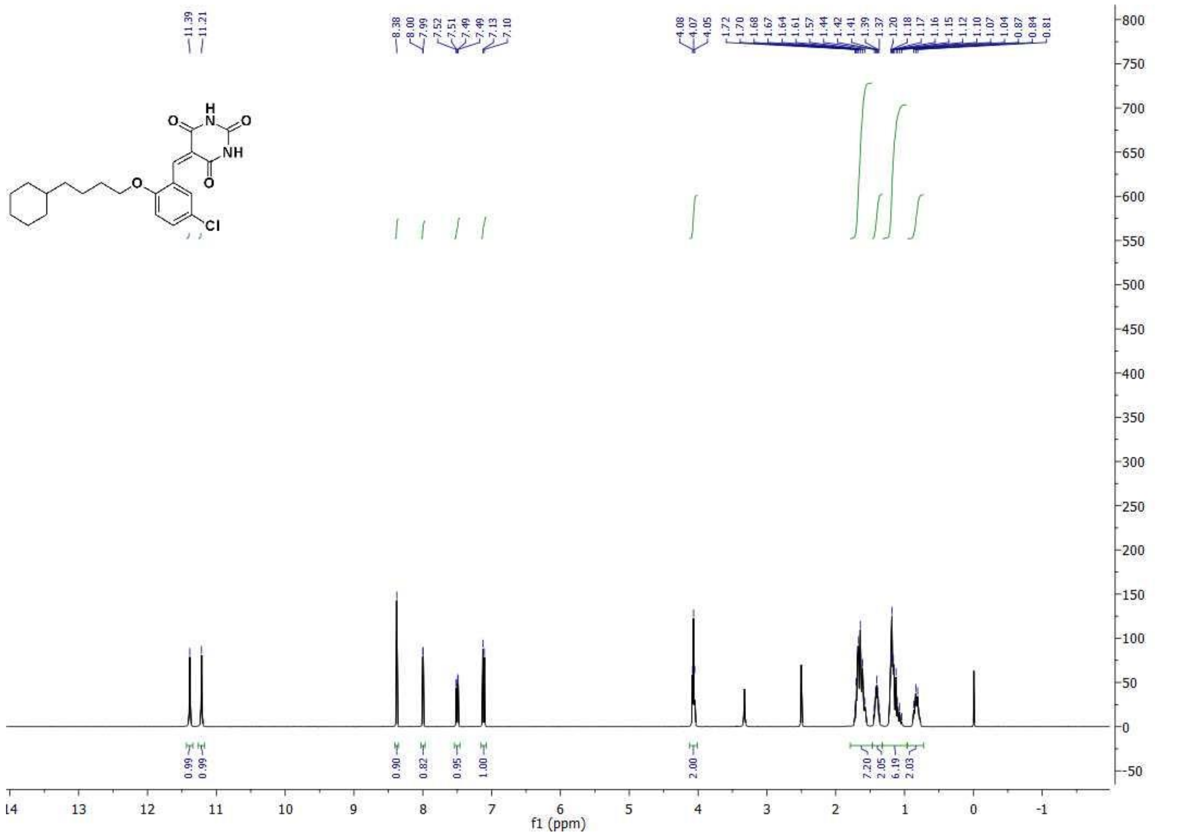

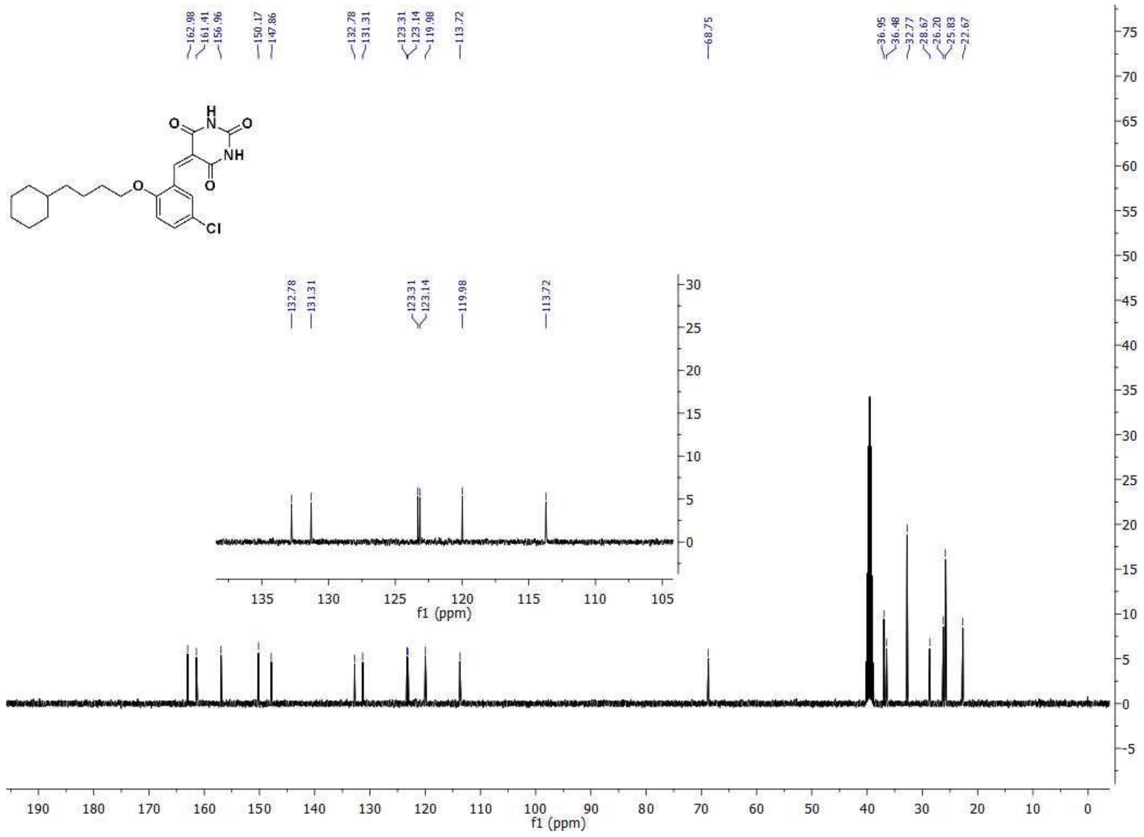

6

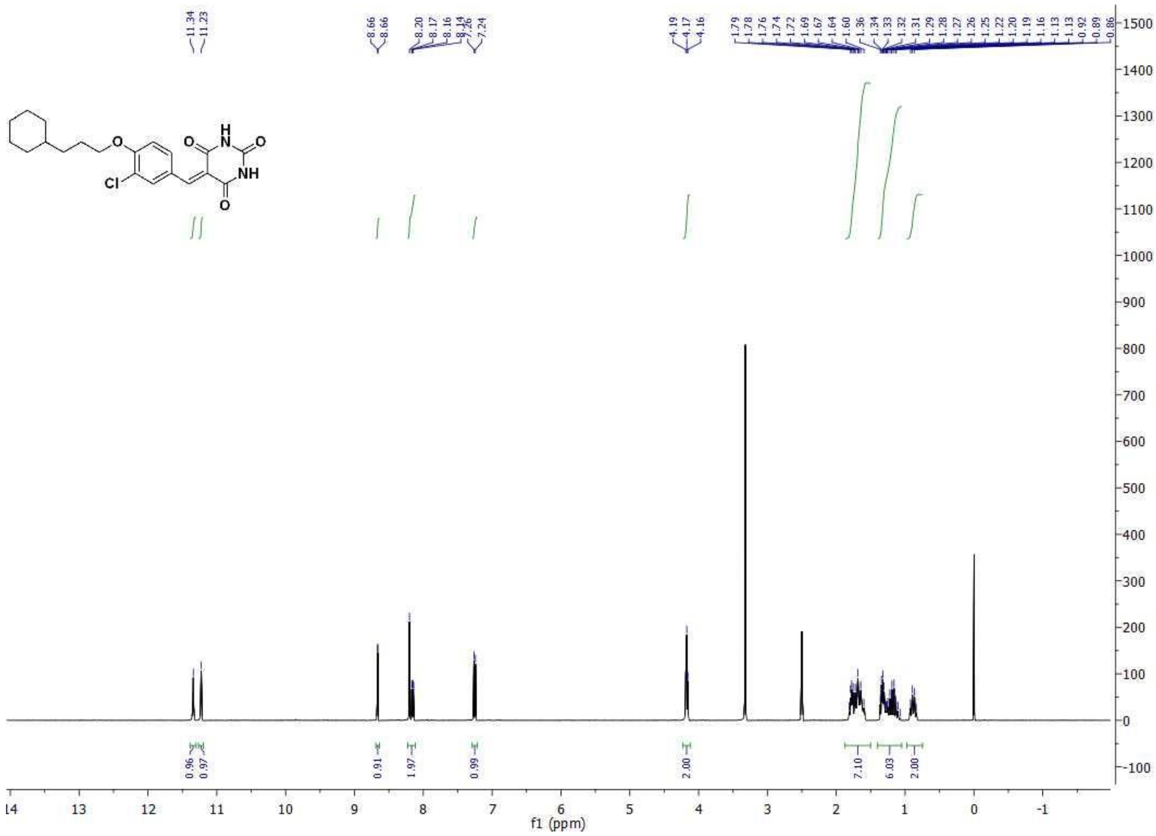

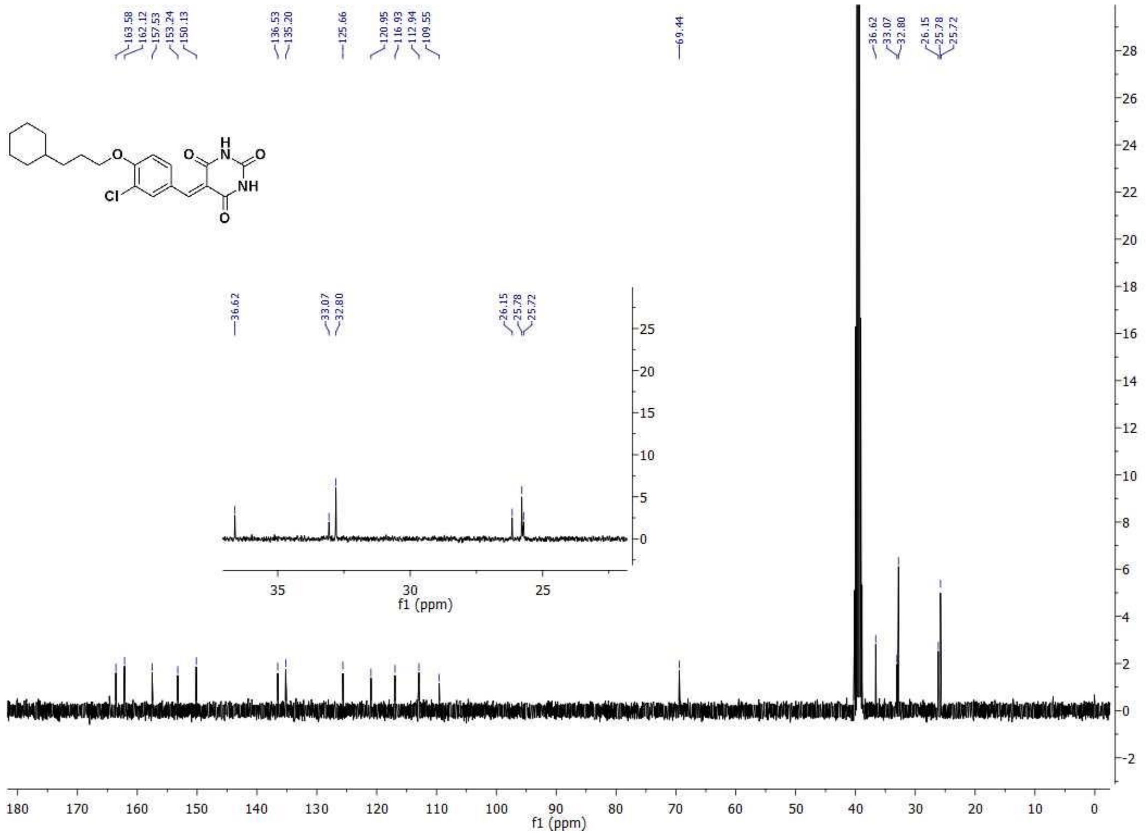

6. References

1 The PyMOL Molecular Graphics System, Version 1.8 Schrödinger, LLC.

2 Sjogren, T. *et al.* Crystal structure of microsomal prostaglandin E2 synthase provides insight into diversity in the MAPEG superfamily. *Proc. Natl. Acad. Sci. U. S. A.* **110**, 3806-3811, doi:10.1073/pnas.1218504110 (2013).

3 Li, D. *et al.* Crystallizing Membrane Proteins in the Lipidic Mesophase. Experience with Human Prostaglandin E2 Synthase 1 and an Evolving Strategy. *Crystal growth & design* **14**, 2034-2047, doi:10.1021/cg500157x (2014).

4 Consortium, U. The universal protein resource (UniProt). *Nucleic Acids Res.* **36**, D190-D195 (2008).

5 UniProt, C. The universal protein resource (UniProt). *Nucleic Acids Res* **36**, D190-195, doi:10.1093/nar/gkm895 (2008).

6 Sali, A., Potterton, L., Yuan, F., van Vlijmen, H. & Karplus, M. Evaluation of comparative protein modeling by MODELLER. *Proteins* **23**, 318-326, doi:10.1002/prot.340230306 (1995).

7 Nayeem, A., Sitkoff, D. & Krystek, S., Jr. A comparative study of available software for high-accuracy homology modeling: from sequence alignments to structural models. *Protein Sci* **15**, 808-824, doi:10.1110/ps.051892906 (2006).

8 Studio, D. version 2.5. *Accelrys Inc.: San Diego, CA, USA* (2009).

9 Shen, M. y. & Sali, A. Statistical potential for assessment and prediction of protein structures. *Protein Science* **15**, 2507-2524 (2006).

10 Morris, G. M. *et al.* AutoDock4 and AutoDockTools4: Automated docking with selective receptor flexibility. *J. Comput. Chem.* **30**, 2785-2791 (2009).

11 Faragher, R. J. & Schwan, A. L. New deuterated oligo(ethylene glycol) building blocks and their use in the preparation of surface active lipids possessing labeled hydrophilic tethers. *J. Org. Chem.* **73**, 1371-1378, doi:10.1021/jo701979z (2008).

12 Saari, W. S., Schwering, J. E., Lyle, P. A., Smith, S. J. & Engelhardt, E. L. Cyclization-activated prodrugs. Basic esters of 5-bromo-2'-deoxyuridine. *J. Med. Chem.* **33**, 2590-2595 (1990).

13 Baron, R. *et al.* Hydrogen-bonded CdS nanoparticle assemblies on electrodes for photoelectrochemical applications. *Angew. Chem. Int. Ed. Engl.* **44**, 4010-4015, doi:10.1002/anie.200463055 (2005).

14 Hidalgo-Figueroa, S. *et al.* Discovery of thiazolidine-2,4-dione/biphenylcarbonitrile hybrid as dual PPAR alpha/gamma modulator with antidiabetic effect: in vitro, in silico and in vivo approaches. *Chem. Biol. Drug Des.* **81**, 474-483, doi:10.1111/cbdd.12102 (2013).

15 Hamza, A. *et al.* Understanding microscopic binding of human microsomal prostaglandin E synthase-1 (mPGES-1) trimer with substrate PGH2 and cofactor GSH: insights from computational alanine scanning and site-directed mutagenesis. *J. Phys. Chem. B* **114**, 5605-5616, doi:10.1021/jp100668y (2010).

16 Huang, X. *et al.* Structural and functional characterization of human microsomal prostaglandin E synthase-1 by computational modeling and site-directed mutagenesis. *Bioorg. Med. Chem.* **14**, 3553-3562, doi:10.1016/j.bmc.2006.01.010 (2006).

17 Harding, L., Wang, Z. & Tai, H. H. Stimulation of prostaglandin E2 synthesis by interleukin-1beta is amplified by interferons but inhibited by interleukin-4 in human amnion-derived WISH cells. *Biochim. Biophys. Acta* **1310**, 48-52 (1996).
